# Interfacial Molecular Mechanisms Governing the NMR Relaxation of Clay-Bound Water in Organic-Rich Shales with Implications for NMR Logging

**DOI:** 10.3390/molecules31183185

**Published:** 2026-09-10

**Authors:** Xuanhua Zhang, Xinmin Ge, Zhenying Liu, Minjie Li

**Affiliations:** 1 School of Geosciences, China University of Petroleum (East China), Qingdao 266580, China; 2 CNPC Key Well Logging Laboratory, Qingdao 266580, China; 3 Laboratory for Marine Mineral Resources, Qingdao National Laboratory for Marine Science and Technology, Qingdao 266071, China; 4 No. 3 Oil Production Plant of PetroChina Qinghai Oilfield Company, Haixi 816400, China; 5 No. 2 Gas Production Plant of China National Petroleum Corporation Changqing Oilfield Branch, Yulin 719000, China

**Keywords:** clay-bound water, shale surface chemistry, interfacial molecular interactions, molecular dynamics simulation, paramagnetic centers, surface relaxivity, low-field nuclear magnetic resonance

## Abstract

Organic-rich shale contains chemically heterogeneous mineral–organic interfaces that produce strong and spatially variable proton surface relaxation, complicating the identification of clay-bound water and the conversion of NMR relaxation time into pore size. Conventional interpretations commonly treat surface relaxivity as a constant, while the respective contributions of water-retaining surface chemistry, molecular restriction, and paramagnetic centers remain insufficiently separated. In this study, Wufeng–Longmaxi shale samples from the Yongchuan Block were investigated using mineralogical and pore structural characterization, Fourier transform infrared spectroscopy, X-ray photoelectron spectroscopy, cation exchange capacity, zeta potential, electron paramagnetic resonance, controlled hydration, one- and two-dimensional low-field time domain NMR, and molecular dynamics simulations. Under the present fluid and acquisition conditions, strongly surface-associated water was operationally identified mainly at T_2_ < 1.6 ms and T_1_ < 85 ms, while the effective transverse surface relaxivity ranged from 3.6 to 9.1 μm/s. Water retention was more closely associated with cation exchange capacity and surface –OH/O–C environments, whereas relaxation efficiency was controlled more directly by EPR-detectable paramagnetic centers and restricted molecular motion of interfacial water. Simulations of Na-smectite, chlorite, illite, and kerogen-covered illite revealed systematic differences in water density layering, adsorption strength, hydrogen bond persistence, molecular residence, translational diffusion, rotational reorientation, and proton–proton dipolar correlation. A chemistry-informed model combining the EPR-derived paramagnetic center density with a surface area-weighted molecular restriction index explained 86% of the measured relaxivity variation, with an adjusted R^2^ of 0.83 and leave-one-out cross-validation RMSE and MAE values of 0.71 and 0.57 μm/s, respectively. At the core scale, the variable relaxivity interpretation reduced the mean absolute percentage error of characteristic pore diameter from 21.9% to 4.5% and the RMSE of the clay-bound water fraction from 3.4 to 0.4 percentage points relative to the fixed relaxivity method. Transfer to NMR logging further reduced lithology-dependent biases in pore size conversion and clay-bound water partitioning. These results define shale surface relaxivity as an emergent interfacial property arising from coupled magnetic and molecular controls and provide a mechanistic basis for NMR analysis of chemically heterogeneous shale materials.

## 1. Introduction

Low-field time-domain nuclear magnetic resonance (NMR) is increasingly used to characterize hydrogen-bearing components and pore fluid distributions in unconventional shale because it is nondestructive, sensitive to molecular mobility, and applicable to both laboratory core analysis and downhole measurements. Song and Kausik [1] summarized the development of NMR as an important tool for investigating unconventional porous media, while Li et al. [2] demonstrated that shale water mobility is closely associated with nanopore confinement and surface interactions. In organic-rich shale, however, the measured signal may contain contributions from clay-bound water, capillary-confined water, mobile pore water, hydrocarbons, kerogen, and solid bitumen. Zhang et al. [3] showed that clay minerals, kerogen, and hydrocarbons have distinguishable but partially overlapping one- and two-dimensional relaxation responses. Zhou et al. [4] similarly demonstrated that mineral phases and organic matter can contribute to the short relaxation region of gas shale spectra. Although integrated NMR workflows have improved liquid saturation and fluid-component interpretation in source rocks [5], the assignment of short T_2_ signals remains uncertain because relaxation is influenced simultaneously by pore dimensions, surface chemistry, molecular mobility, and local magnetic environments [6].

Recent studies have attempted to distinguish shale water populations through controlled saturation, drying, heating, centrifugation, and multidimensional relaxation measurements. Yang and Yu [7] found that water mobility in shale nanopores depends strongly on the degree of confinement and the applied pressure gradient. Jin et al. [8] developed an NMR-based approach for quantitatively separating bound and movable fluids in porous rocks. Silletta et al. [9] introduced a pulse sequence that improved the identification of solid organic matter on T_1_–T_2_ maps, whereas Li et al. [10] demonstrated that two-dimensional NMR can characterize the occurrence of oil and water in preserved shale cores. Fleury et al. [11] further linked porewater content and mobility to the pore architecture of clay-rich rocks. Fan et al. [12] established a T_1_–T_2_-based fluid classification method for lacustrine shale, and Gu et al. [13] quantified fluid saturation using two-dimensional relaxation distributions. These studies have substantially improved the separation of bound water, movable water, hydrocarbons, and solid organic signals. Nevertheless, most classifications still rely on empirically selected T_2_ cutoffs or predefined regions on T_1_–T_2_ maps. Such boundaries are not necessarily transferable among formations because mineral assemblage, organic matter coverage, brine chemistry, temperature, resonance frequency, and echo spacing can all modify the relaxation response. Consequently, identical relaxation times do not necessarily represent identical pore sizes or water states in chemically heterogeneous shale.

A major source of this non-uniqueness is the common assumption that transverse surface relaxivity, ρ_2_, is uniform within a sample or formation. Yuan and Rezaee [14] demonstrated that Fe-bearing paramagnetic minerals can markedly shorten T_2_ and shift NMR-converted pore size distributions. Livo et al. [15] further showed that surface relaxivity depends not only on the abundance of paramagnetic minerals but also on their spatial distribution and accessibility to pore fluids. Elsayed et al. [16] reported progressively shorter transverse relaxation times with increasing clay content, whereas Ge et al. [17] showed that clay-rich shale may exhibit complex transverse relaxation under inhomogeneous internal magnetic fields. For deep Wufeng–Longmaxi shale, Wang et al. [18] confirmed that low-field NMR parameters vary systematically with mineral composition and pore structure. Cheng et al. [19] subsequently determined sample-dependent shale surface relaxivity values and emphasized that a fixed value may generate substantial errors during pore size conversion. Zhang et al. [20] further showed that adsorbed, bound, and movable water occupy different pore domains and respond differently to clay minerals and organic matter. These findings establish that mineralogical composition affects NMR relaxation, but bulk clay or pyrite abundance alone cannot distinguish the contributions of accessible paramagnetic sites, surface charge, oxygen-containing functional groups, molecular confinement, and internal magnetic field gradients. A direct experimental and molecular separation of these controls is therefore required.

Water sorption measurements and molecular simulations provide a complementary route for resolving the physicochemical state of interfacial water. Chen et al. [21] showed that water uptake in overmature Wufeng–Longmaxi shale varies with mineral composition, organic matter, and temperature. Lawal et al. [22] demonstrated that kerogen maturity modifies kerogen–water interactions and water affinity at the molecular scale. Underwood and Bourg [23] used large-scale molecular dynamics simulations to reveal structural changes in hydrated smectite during progressive dehydration, while Shen and Bourg [24] quantified the distance-dependent interaction between smectite nanoparticles in water. Jia et al. [25] characterized the association and hydration behavior of illite particles, and Shi et al. [26] demonstrated that different shale clay minerals exhibit distinct surface–water interactions and wettability. Sanchouli et al. [27] further showed that kerogen surface structure, flexibility, and heteroatom distribution regulate water adsorption and hydrogen bonding. Jiang et al. [28] applied molecular dynamics simulations to examine pressure- and wettability-dependent water imbibition in shale nanopores. Collectively, these studies demonstrate that mineral identity and organic coverage influence water density layering, adsorption strength, hydrogen bond persistence, residence time, translational diffusion, and rotational mobility. However, most previous simulations have focused on adsorption, swelling, wettability, or transport. Molecular descriptors of interfacial water restriction have rarely been quantitatively linked to experimentally measured proton surface relaxation, particularly in combination with independently measured paramagnetic center density.

The organic-rich Wufeng–Longmaxi shale provides a suitable material system for examining this unresolved relationship. Gao et al. [29] demonstrated that water occurrence in ultra-deep Wufeng–Longmaxi shale is controlled jointly by pore size, clay minerals, and organic matter. He et al. [30] further showed that its lithofacies and physical properties vary systematically with mineral assemblage, total organic carbon, and pore structure. Shale pore systems span multiple length scales and are commonly characterized using complementary techniques, including SEM or X-ray computed tomography for direct pore imaging, low-pressure N_2_/CO_2_ adsorption and mercury intrusion porosimetry for pore size and surface area characterization, and NMR for nondestructive evaluation of fluid-filled pore space. Because these methods differ in spatial resolution and probe accessibility, their combined use is generally required to constrain the heterogeneous pore structure of shale. In the present study, gas adsorption was used primarily to characterize pore geometry and accessible surface area, whereas NMR was employed to probe water-filled pore space and relaxation behavior, for which pore size conversion additionally depends on surface relaxivity [6,18,31,32]. On this basis, the present study investigated whether variations in clay-bound water relaxation could be explained by the coupled effects of paramagnetic enhancement and interfacial molecular restriction. Shale samples from the Yongchuan Block were characterized using X-ray diffraction, gas adsorption, Fourier transform infrared spectroscopy, X-ray photoelectron spectroscopy, cation exchange capacity and zeta-potential measurements, electron paramagnetic resonance, controlled hydration, one- and two-dimensional low-field time-domain NMR, and molecular dynamics simulations of representative mineral–water and organic-covered interfaces. The specific objectives were to: (i) identify the relaxation domain of strongly surface-associated water using progressive hydration rather than an externally imposed universal cutoff; (ii) distinguish the respective roles of water-retaining surface chemistry, EPR-detectable paramagnetic centers, and molecular restriction in controlling NMR relaxation; and (iii) establish a chemistry-informed variable relaxivity model and evaluate its transfer from core-scale analysis to NMR logging interpretation. The primary novelty of this study lies in establishing a chemistry-informed variable relaxivity framework that quantitatively integrates the independently measured EPR-derived paramagnetic center density with a molecular dynamics-derived restriction index to explain sample-specific NMR surface relaxivity. The subsequent transfer to NMR logging is treated as a scale validation and application of this framework rather than as the primary methodological novelty.

## 2. Results and Discussion

### 2.1. Mineralogical, Pore Structural, and Interfacial Chemical Characteristics

#### 2.1.1. Mineral and Organic Matter Composition

**Figure 1:**
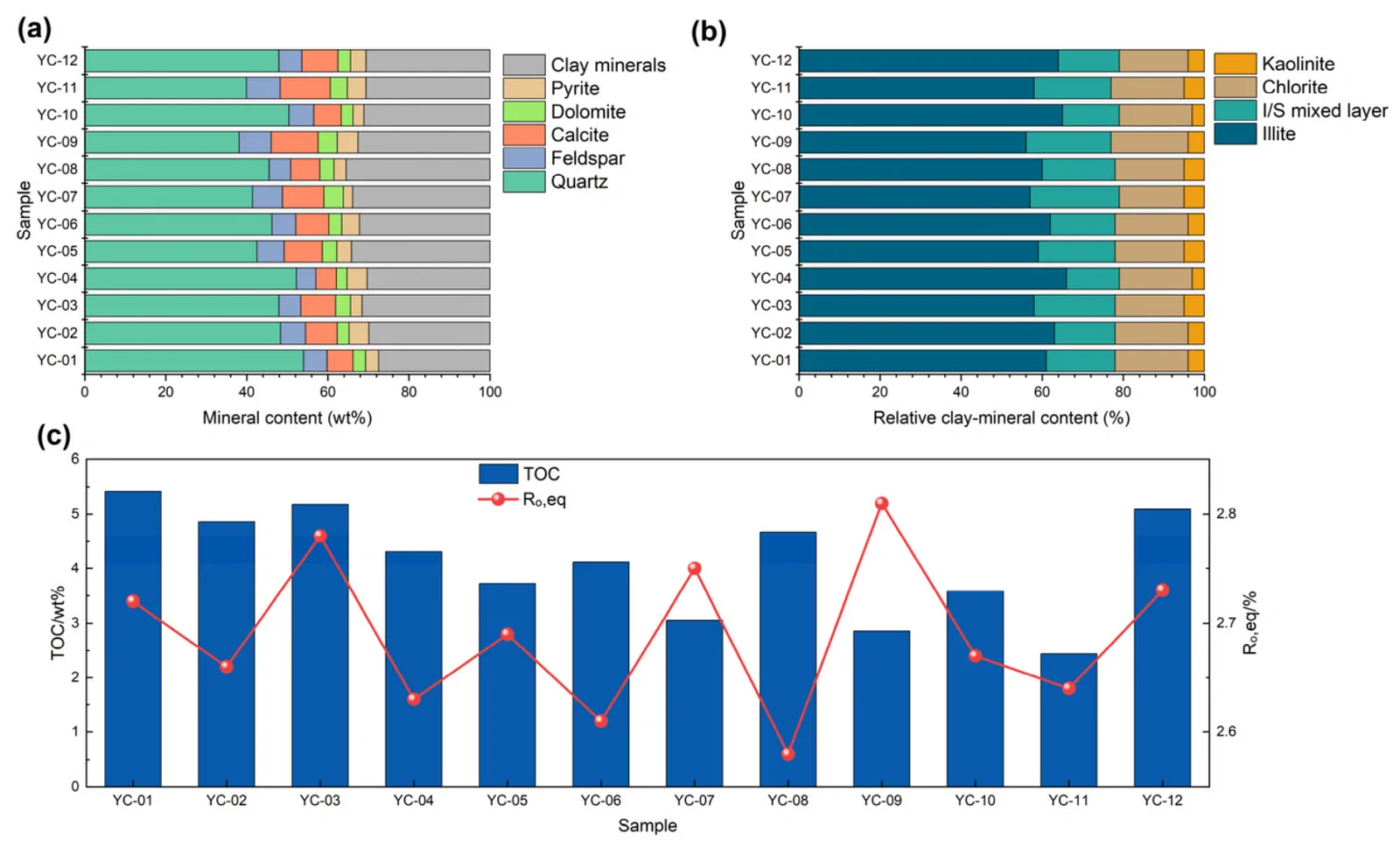
Mineralogical and organic matter characteristics of the Wufeng–Longmaxi shale samples from the Yongchuan Block: (**a**) whole-rock mineral composition; (**b**) clay mineral composition normalized to the total clay fraction; and (**c**) total organic carbon content and equivalent vitrinite reflectance, *R*_o,eq_.

Whole-rock X-ray diffraction showed that the investigated shales were dominated by quartz and clay minerals, with subordinate carbonate minerals, feldspar, and pyrite (Figure 1a). Quartz contents ranged from 38.1 to 54.0 wt%, with an average of 46.2 wt%, whereas total clay mineral contents varied from 27.5 to 35.5 wt% and averaged 31.7 wt%. Calcite and dolomite together accounted for 7.7–16.6 wt%, while feldspar and pyrite contents were 4.8–8.3 wt% and 2.3–5.1 wt%, respectively. The mineral assemblages therefore represent quartz-dominated to mixed siliceous–argillaceous compositions rather than clay-rich end members, broadly consistent with previously reported organic-rich Wufeng–Longmaxi shales [18,29,30]. The relatively restricted range of total clay content, combined with more apparent variations in pyrite and organic matter abundance, provides useful compositional contrasts for evaluating whether the subsequent NMR response is controlled solely by pore geometry or additionally by the physicochemical properties of the exposed interfaces.

Illite was the dominant clay mineral, accounting for 56–66% of the clay fraction, followed by chlorite at 16–19%, mixed-layer illite/smectite (I/S) at 13–22%, and kaolinite at 3–5% (Figure 1b). The predominance of illite and chlorite, together with the limited I/S and kaolinite contents, is consistent with the advanced burial and diagenetic evolution of the Wufeng–Longmaxi shale. Nevertheless, total clay content does not represent a chemically uniform water–solid interface. Mixed-layer I/S contains exchangeable interlayer sites with comparatively strong hydration affinity, whereas illite provides charged basal and edge surfaces with more limited interlayer accessibility. Chlorite additionally contains hydroxyl-bearing and Fe-bearing structural environments that may influence both water retention and local magnetic relaxation. Samples containing similar total amounts of clay may therefore display different interfacial water populations and relaxation efficiencies because their clay mineral proportions and accessible surface sites differ.

Total organic carbon contents ranged from 2.44 to 5.42 wt%, with an average of 4.11 wt%, confirming that the samples were obtained from the organic-rich interval of the formation (Figure 1c). The equivalent vitrinite reflectance, *R*_o,eq_, varied within a relatively narrow range of 2.58–2.81%, averaging 2.69%, and indicated uniformly overmature organic matter. The limited maturity variation reduces the likelihood that differences in thermal evolution constitute the principal source of the observed intersample NMR variability. At this maturity stage, however, TOC should not be interpreted as being directly proportional to the detectable proton population because extensive hydrocarbon generation and secondary cracking have substantially reduced the hydrogen content of the residual organic matter. Instead, organic matter is expected to influence the water response mainly through organic-hosted microporosity and partial coverage of hydrophilic mineral surfaces.

The sample set contains several representative compositional contrasts. YC-01 is relatively quartz-rich and organic-rich, containing 54.0 wt% quartz, 27.5 wt% clay minerals, and 5.42 wt% TOC. YC-08 has a comparable TOC content of 4.67 wt% but the highest total clay content of 35.5 wt%. In contrast, YC-09 contains 32.6 wt% clay minerals and the highest pyrite content of 5.1 wt%, while its TOC content is only 2.86 wt%. These contrasts partially decouple the abundances of clay minerals, organic matter, and Fe-bearing phases and therefore provide a suitable basis for subsequent comparisons with pore structure, surface chemistry, EPR response, and NMR relaxation. At this stage, however, the bulk mineralogical data define only the compositional framework; the actual water accessibility and proton relaxation efficiency of these mineral and organic interfaces require the surface-specific measurements presented in Section 2.1.2 and Section 2.1.3. The complete whole-rock mineral, clay mineral, TOC, and *R*_o,eq_ data for YC-01–YC-12 are provided in Table S1.

#### 2.1.2. Pore Structure and Specific Surface Characteristics

**Figure 2:**
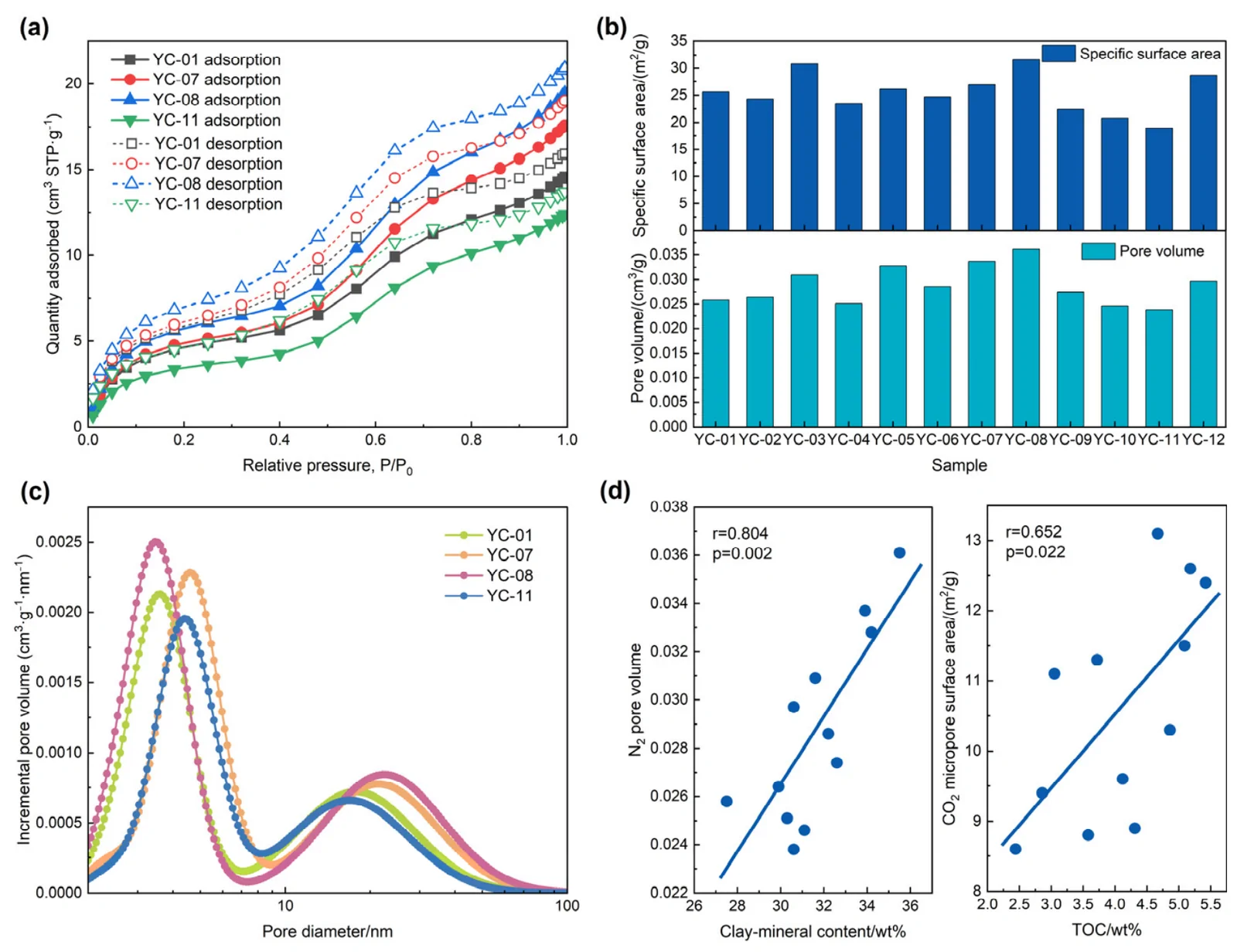
Pore structural characteristics of the Wufeng–Longmaxi shale samples from the Yongchuan Block: (**a**) N_2_ adsorption–desorption isotherms of representative samples; (**b**) N_2_-BET specific surface area and accessible pore volume; (**c**) N_2_-derived mesopore size distributions; and (**d**) relationships of pore volume and micropore surface area with clay mineral content and TOC, respectively.

The low-pressure N_2_ adsorption–desorption isotherms exhibit a type IV response accompanied by a transitional H3–H4 hysteresis loop (Figure 2a). Adsorption increases gradually at low relative pressures and becomes more pronounced above P/P_0_ ≈ 0.45, indicating capillary condensation within mesopores. A further increase near saturation reflects contributions from relatively large interparticle voids and microfracture-associated spaces. The hysteresis characteristics are compatible with a pore system containing slit-like and constricted pores, although pore geometry cannot be uniquely determined from the loop shape alone. The broadly similar isotherm morphology suggests that the samples share a common pore network framework, whereas differences in adsorption capacity indicate appreciable variations in gas-accessible surface area and pore volume.

The N_2_-BET specific surface area ranges from 18.9 to 31.6 m^2^/g, with an average of 25.4 m^2^/g, while the N_2_-accessible pore volume varies from 0.0238 to 0.0361 cm^3^/g and averages 0.0287 cm^3^/g (Figure 2b and Table S2). YC-08 exhibits both the largest surface area and pore volume, whereas YC-11 shows the lowest values. Surface area and pore volume do not, however, vary proportionally throughout the sample set. For example, YC-07 has a relatively large pore volume of 0.0337 cm^3^/g but an intermediate surface area of 26.9 m^2^/g, whereas YC-03 has a larger surface area of 30.8 m^2^/g with a slightly smaller pore volume of 0.0309 cm^3^/g. This divergence indicates that pore volume is influenced more strongly by mesopores and larger voids, whereas a substantial proportion of the measurable surface area is contributed by smaller pores.

The N_2_-derived pore size distributions are dominated by pores of approximately 2.5–8.0 nm, with principal maxima at 3.4–4.6 nm (Figure 2c). Broader shoulders extending from approximately 10 to 30 nm contribute appreciably to pore volume but less substantially to total surface area. CO_2_ adsorption additionally resolves a micropore population concentrated mainly at 0.55–0.75 nm. The CO_2_-DFT micropore surface area ranges from 8.6 to 13.1 m^2^/g, while the corresponding micropore volume varies from 0.0037 to 0.0053 cm^3^/g. Although these micropores account for only a limited proportion of total pore volume, they provide a comparatively large solid–fluid contact area and may therefore contribute disproportionately to water adsorption and surface-influenced proton relaxation. Comparable multiscale pore systems have been reported for organic-rich Wufeng–Longmaxi shale, in which clay-associated mesopores and organic matter-hosted micropores jointly control fluid storage and interfacial area [20,21,29,30]. The complete N_2_ and CO_2_ adsorption-derived pore structure parameters for YC-01–YC-12 are provided in Table S2.

The N_2_-accessible pore volume is positively correlated with total clay mineral content (r = 0.804, *p* = 0.002; Figure 2d), consistent with a contribution from clay-associated interparticle, intercrystalline, and slit-like pores. In contrast, the relationship between total clay content and BET surface area is weaker and statistically insignificant (r = 0.385, *p* = 0.216). BET surface area shows a moderate positive relationship with TOC (r = 0.649, *p* = 0.022), and a similar relationship is observed between TOC and CO_2_-derived micropore surface area (r = 0.652, *p* = 0.022). These correlations suggest that organic matter-hosted micropores contribute to the measurable surface area, whereas clay-associated pore systems make a larger contribution to accessible pore volume. Nevertheless, because the dataset contains only 12 samples and mineralogical variables are not fully independent, these relationships should be interpreted as compositional trends rather than definitive evidence of single-component control.

The pore structure results establish the geometric basis for the subsequent relaxation analysis. Samples with comparable pore volumes may expose different surface areas, whereas samples with similar BET surface areas may contain different proportions of mineral and organic interfaces. Moreover, the N_2_- and CO_2_-derived parameters represent gas probe-accessible pore surfaces and do not necessarily equal the surface area contacted by saline water under the NMR conditions. Accordingly, these measurements are used to constrain relative variations in pore geometry and accessible interface rather than being treated directly as a complete measure of water-accessible S/V. The chemical identity, charge properties, organic coverage, and paramagnetic activity of the accessible surfaces are examined in Section 2.1.3.

#### 2.1.3. Surface Functional Groups, Charge Properties, and Paramagnetic Centers

**Figure 3:**
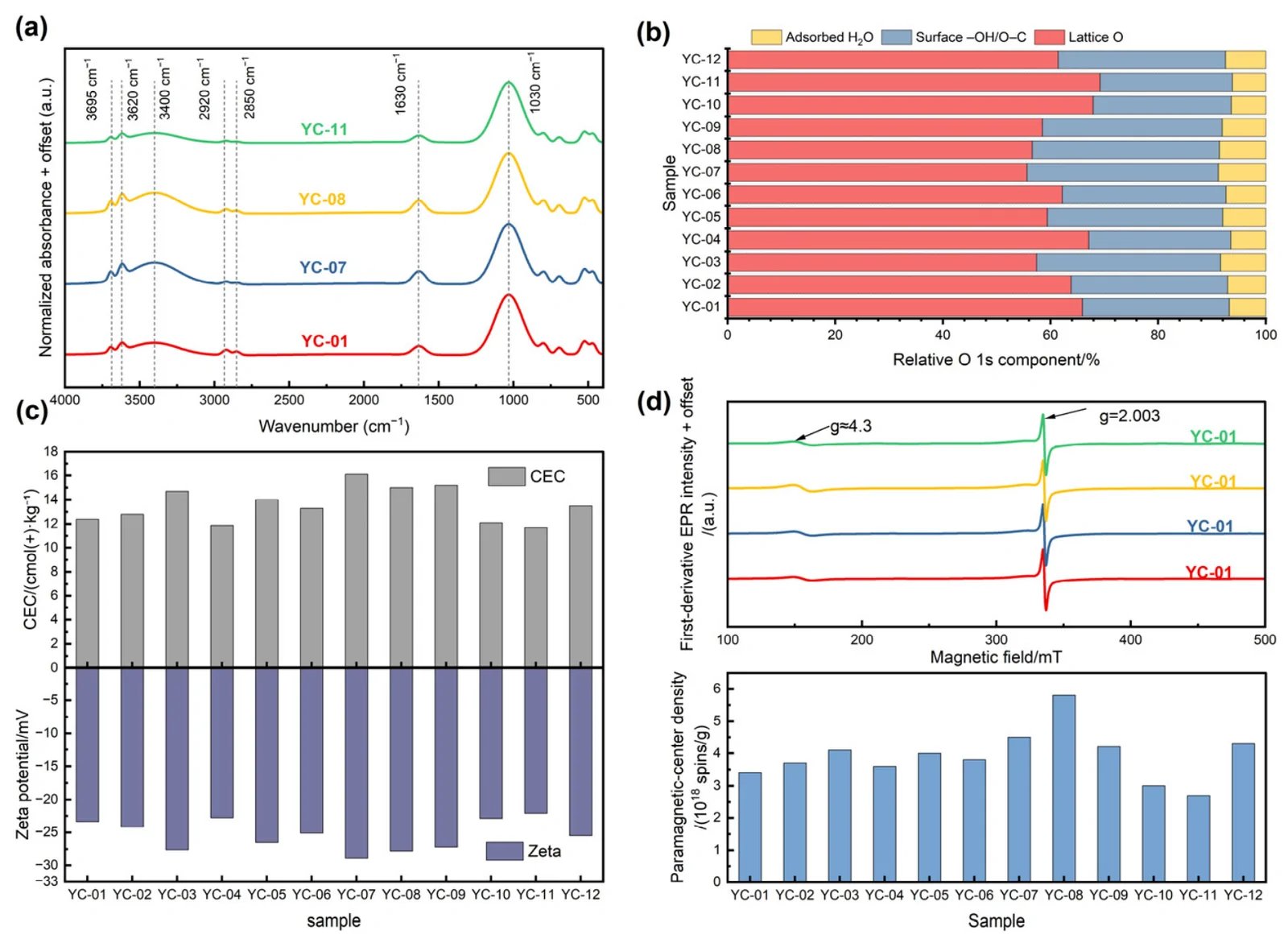
Interfacial chemical, electrokinetic, and magnetic characteristics of the investigated shale samples: (**a**) ATR–FTIR spectra of representative samples; (**b**) relative contributions of the fitted O 1s components obtained from XPS analysis; (**c**) cation exchange capacity and zeta potential; and (**d**) representative EPR spectra and effective EPR-detectable paramagnetic center densities.

The ATR–FTIR spectra of the representative samples are dominated by the Si–O stretching envelope centered near 1030 cm^−1^, together with structural hydroxyl bands at 3620–3695 cm^−1^ (Figure 3a). The broad feature between 3200 and 3500 cm^−1^ is attributable mainly to hydrogen-bonded surface hydroxyls and adsorbed water, whereas the band near 1630 cm^−1^ contains a substantial H–O–H bending contribution and may partly overlap with aromatic C=C and oxygen-containing organic vibrations. The weak bands at approximately 2920 and 2850 cm^−1^ indicate a limited abundance of aliphatic C–H groups, consistent with the uniformly overmature organic matter. The relative intensity of the hydroxyl- and water-associated envelope differs among the representative samples, with YC-08 displaying a more pronounced broad absorption than YC-01. Because the spectra were normalized to the Si–O band, however, these variations are interpreted as relative differences in oxygen-bearing interfacial environments rather than as direct measurements of absolute hydroxyl-site density.

High-resolution O 1s spectra further separate lattice oxygen, a combined surface –OH/O–C component, and a high-binding-energy component assigned to weakly bound interfacial oxygen species, including molecularly adsorbed water (Figure 3b). The surface –OH/O–C component accounts for 24.6–35.6% of the fitted O 1s envelope. Its relative abundance generally increases with clay mineral content and the proportion of mixed-layer illite/smectite, but the relationship is not proportional across the sample set, indicating additional influences from clay type, organic-surface coverage, and probe accessibility. Notably, YC-01 has a relatively large gas-accessible surface area but a smaller exposed –OH/O–C fraction than YC-08. This contrast is consistent with partial masking of hydrophilic mineral sites by organic matter, although XPS and gas adsorption probe different surface populations and therefore should not be treated as equivalent measurements of water-accessible interfacial area. Comparable compositional effects on water affinity have been reported for overmature Wufeng–Longmaxi shale [21].

The cation exchange capacity ranges from 11.7 to 16.1 cmol(+)·kg^−1^, while the zeta potential measured in 10 mmol/L NaCl solution at pH 7 varies from −22.1 to −28.9 mV (Figure 3c). YC-07 exhibits both the highest cation exchange capacity and the most negative zeta potential, whereas YC-11 shows the lowest cation exchange capacity and the least negative zeta potential. Cation exchange capacity quantifies the abundance of exchangeable charge sites, whereas zeta potential reflects the electrokinetic response of dispersed particles under the specified fluid conditions; the two parameters should therefore not be interpreted as interchangeable measurements of surface charge density. Their broadly coherent variation nevertheless indicates that samples enriched in mixed-layer illite/smectite contain more charge-related hydration sites. These sites promote counterion accumulation and stabilize interfacial hydration structures, thereby increasing water retention and molecular residence rather than directly prescribing the proton relaxation rate [23,24,25,26].

The room-temperature EPR spectra contain a narrow feature at g = 2.003, assigned predominantly to persistent organic radicals, together with overlapping Fe-related contributions around g ≈ 2.0 and a broader feature near g ≈ 4.3 associated with isolated high-spin Fe^3+^ in distorted coordination environments (Figure 3d). The calibrated, mass-normalized double integrals yield an effective EPR-detectable paramagnetic center density ranging from 2.7 × 10^18^ to 5.8 × 10^18^ spins/g. This descriptor is not proportional to bulk pyrite abundance: YC-02 contains 4.9 wt% pyrite but exhibits only an intermediate EPR response, whereas YC-08 shows the largest paramagnetic center density at a pyrite content of 3.0 wt%. This decoupling confirms that bulk pyrite content cannot be used as a surrogate for the abundance and distribution of EPR-detectable magnetic centers, consistent with previous shale NMR studies [14,15]. Because the calibrated spin density includes both organic radical and Fe-related contributions and does not directly quantify their accessibility to pore water, it is treated here as an effective magnetic site descriptor. Its relevance to proton relaxation is evaluated independently against the NMR-derived parameters in Section 2.2 and Section 2.4.

Taken together, the interfacial measurements define two related but non-equivalent controls on clay-bound water. Cation exchange capacity, charge-related oxygen environments, and zeta-potential behavior primarily describe water affinity, retention, and the tendency toward molecular restriction, whereas EPR-detectable centers define the potential for magnetic relaxation enhancement. The actual relaxation efficiency therefore depends on the spatial proximity and exchange of retained water with these magnetic sites, together with the molecular mobility of the interfacial water. This distinction provides the basis for separating bound water capacity from relaxation efficiency in the following sections.

### 2.2. NMR Relaxation Behavior of Clay-Bound Water

**Figure 4:**
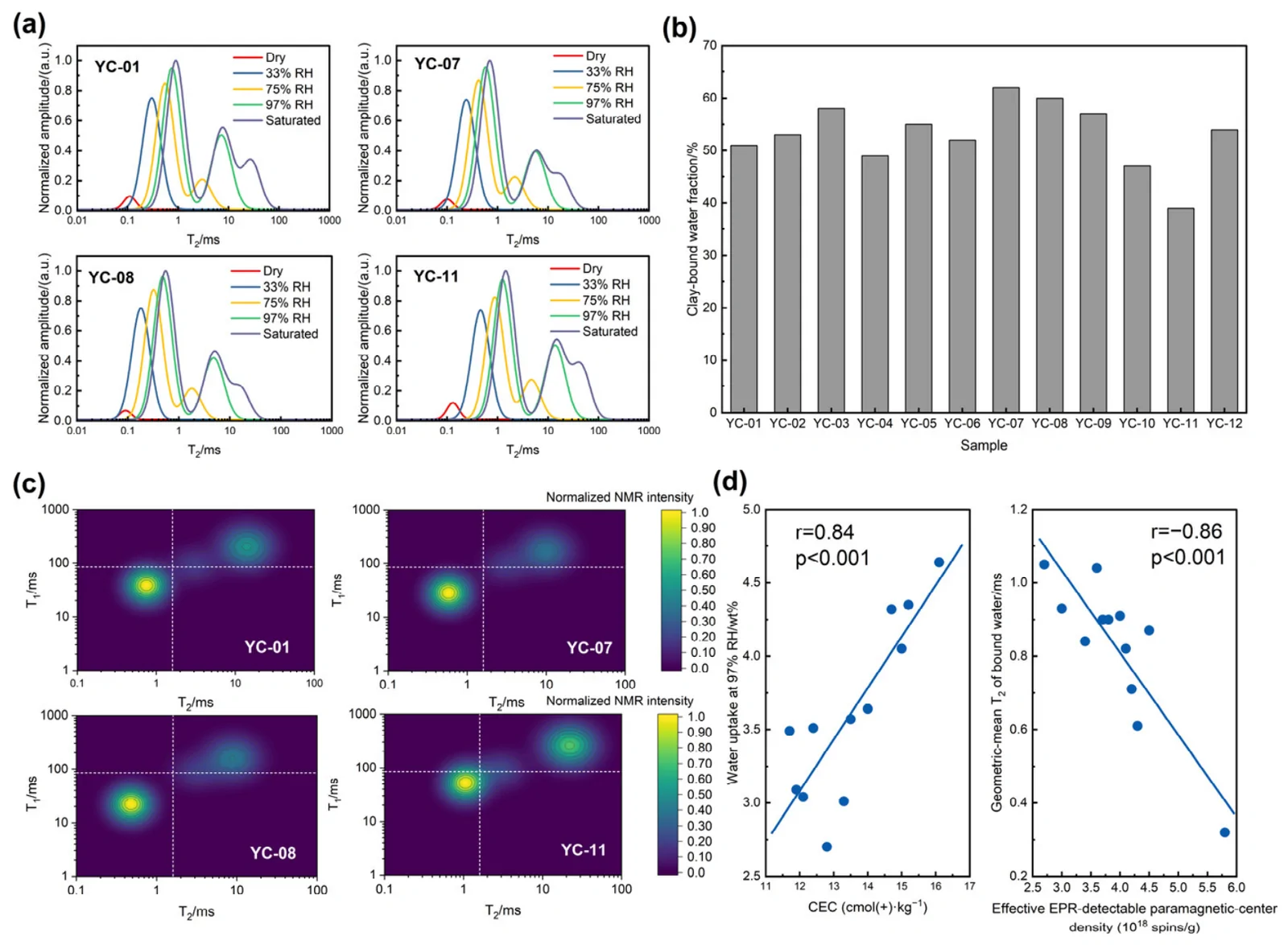
NMR relaxation characteristics of clay-bound water in the investigated shale samples: (**a**) evolution of T_2_ distributions during controlled hydration; (**b**) NMR-defined clay-bound water fractions under vacuum-saturated conditions; (**c**) representative T_1_–T_2_ maps; and (**d**) relationships of water uptake at 97% relative humidity and geometric mean T_2_ of the short relaxation component with cation exchange capacity and effective EPR-detectable paramagnetic center density, respectively.

The dry-state spectra contain only a weak, rapidly decaying background signal, accounting for less than 4% of the fully hydrated NMR amplitude after dead time correction. The subsequent spectral evolution can therefore be attributed predominantly to water introduced during controlled hydration rather than to residual hydrogen in the overmature organic matter. At 33% relative humidity, the T_2_ distributions are dominated by a single short relaxation component centered at 0.18–0.46 ms (Figure 4a). Increasing the relative humidity to 75% shifts this component to 0.32–0.88 ms and produces a weak shoulder at 1.8–4.7 ms. At 97% relative humidity, the spectra become distinctly bimodal, with a persistent short T_2_ population at 0.48–1.25 ms and a broader component between 4.6 and 13.8 ms. A further long relaxation contribution appears after vacuum saturation, indicating progressive occupation of larger pores and pore-center regions. The systematic shift toward longer T_2_ values is consistent with the transition from water directly associated with mineral surfaces to multilayer and capillary water experiencing weaker surface interactions [2,11,20].

The relative contribution of the short T_2_ population differs appreciably among the samples. Based on the controlled hydration calibration and the corresponding T_1_–T_2_ regions, NMR-defined clay-bound water accounts for 39–62% of the total water signal under vacuum-saturated conditions (Figure 4b). YC-07 exhibits the largest clay-bound water fraction, consistent with its high cation exchange capacity and strongly negative zeta potential. YC-08 shows a comparable bound water contribution but a shorter relaxation time, whereas YC-11 contains the smallest bound water fraction and the longest T_2_ within the short relaxation population. These differences indicate that the amount of retained water and its relaxation rate are not governed by the same interfacial property.

The two-dimensional T_1_–T_2_ maps resolve this distinction more clearly (Figure 4c). Strongly surface-associated water is concentrated mainly at T_2_ = 0.2–1.6 ms and T_1_ = 12–85 ms, while the less restricted pore-water population extends toward T_2_ = 8–45 ms and T_1_ = 90–450 ms. These relaxation domains are operational boundaries specific to the present conditions of 12 MHz proton resonance frequency, 0.5 mol/L NaCl brine, 25 ± 0.2 °C, and a CPMG echo spacing of 0.08 ms, rather than intrinsic constants for clay-bound water. Changes in magnetic field strength can alter relaxation spectral density and paramagnetic/internal-gradient contributions, whereas brine composition, temperature, and echo spacing can modify interfacial hydration and molecular mobility or the diffusion-induced attenuation of short T_2_ signals; consequently, the numerical T_1_–T_2_ boundaries should be recalibrated when these conditions change. At a matched water loading of 4.0 wt%, the geometric mean T_2_ of the short relaxation component varies from 0.32 to 1.05 ms across the sample set. This parameter is strongly and negatively correlated with the effective EPR-detectable paramagnetic center density (r = −0.86, *p* < 0.001), whereas water uptake at 97% relative humidity correlates more closely with cation exchange capacity (r = 0.84, *p* < 0.001) (Figure 4d). The former relationship is consistent with the influence of paramagnetic environments on shale proton relaxation reported previously [14,15,19].

The NMR response therefore reflects two coupled but distinguishable interfacial effects. Charged, hydroxyl-rich clay surfaces primarily control how much water is retained and how strongly its molecular motion is restricted, whereas EPR-detectable paramagnetic centers more directly influence the efficiency with which the retained protons relax. Pore size remains important, particularly after multilayer and capillary filling, but it cannot independently account for the marked differences in the short T_2_ component. This separation between water retention capacity and relaxation efficiency provides the experimental basis for evaluating the molecular dynamics of interfacial water and constructing the chemistry-informed surface relaxivity model.

### 2.3. Molecular Structure and Dynamics of Interfacial Water

#### 2.3.1. Interfacial Water Distribution and Adsorption Configuration

**Figure 5:**
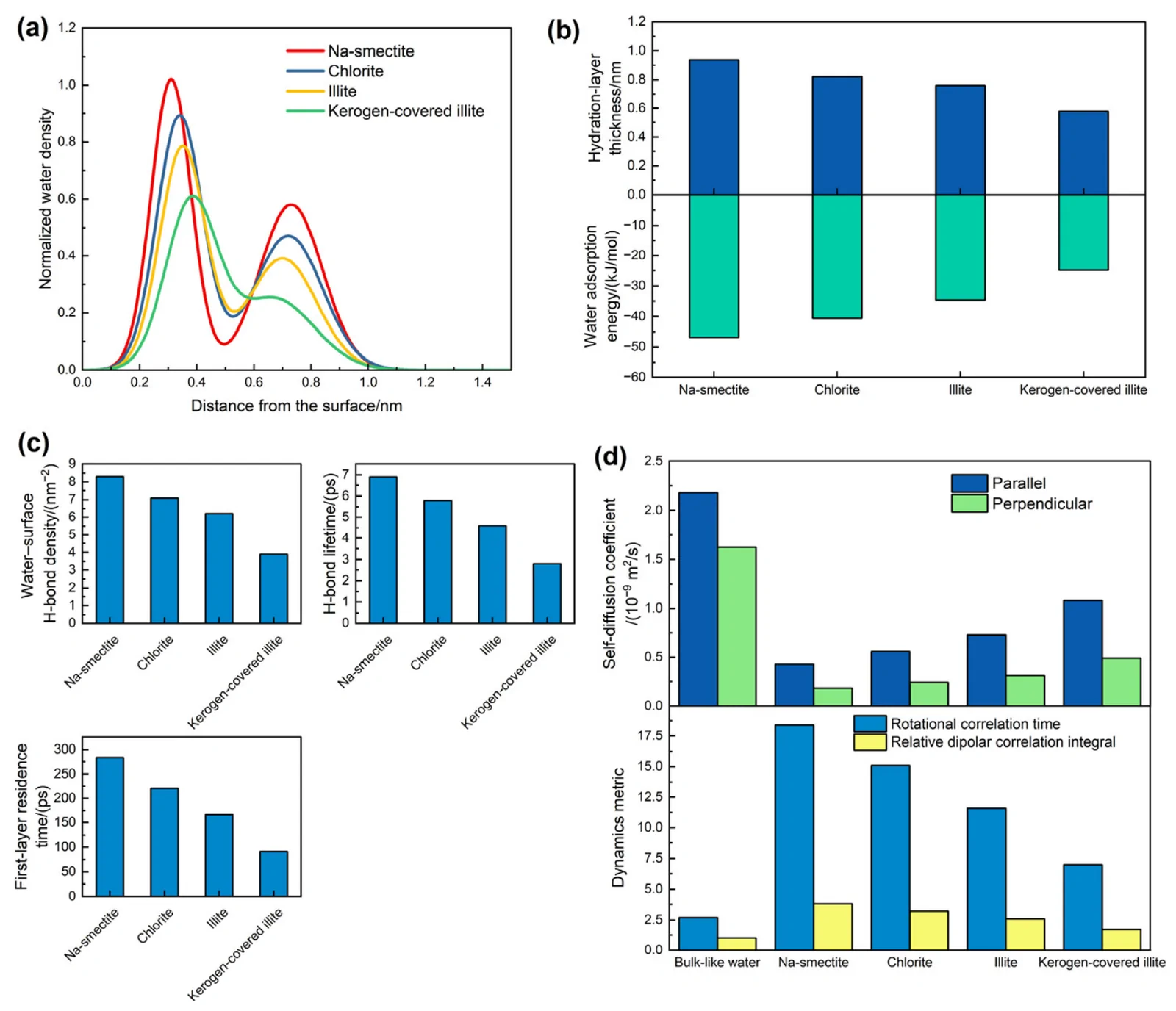
Molecular structure and dynamics of interfacial water at representative shale-related interfaces: (**a**) normalized water density profiles as a function of distance from the surface; (**b**) effective hydration layer thickness and calculated water adsorption energy; (**c**) water–surface hydrogen bond density, hydrogen bond lifetime, and first-layer residence time; and (**d**) self-diffusion coefficients parallel and perpendicular to the interface, rotational correlation time, and relative proton–proton dipolar correlation integral.

Water exhibits pronounced density layering adjacent to all investigated interfaces, although the spatial extent and intensity of the interfacial region differ substantially among the four models (Figure 5a). The first density maximum occurs at 0.24–0.31 nm from the surface and corresponds to water molecules directly coordinated with surface oxygen atoms, hydroxyl groups, or exchangeable cations. A second, broader maximum is resolved at 0.55–0.68 nm. Beyond approximately 0.6–1.0 nm, depending on the interface type, the water density gradually approaches the pore-center value. The effective hydration layer thickness follows the order Na-smectite (0.94 nm) > chlorite (0.82 nm) > illite (0.76 nm) > kerogen-covered illite (0.58 nm).

The Na-smectite model represents the strongly hydratable smectitic component of the mixed-layer illite/smectite identified in the shale samples. Its pronounced water layering arises from the combined effects of negatively charged basal surfaces and hydrated interlayer Na^+^, which generate a compact inner layer followed by a more diffuse secondary hydration shell. Chlorite also maintains a distinct interfacial water structure, although its density perturbation extends over a shorter distance. These differences are consistent with previous molecular simulations showing that clay identity, structural charge, and exchangeable ions regulate surface hydration [23,24,25,26].

Partial kerogen coverage suppresses the first water density maximum and reduces the thickness of the structurally perturbed water region. Under the modeled coverage condition, organic matter therefore does not simply provide an additional adsorption surface but also masks part of the hydrophilic mineral interface. This behavior is consistent with the dependence of kerogen–water interactions on organic structure and heteroatom distribution [22,27], although the magnitude of the effect remains specific to the kerogen structure and surface coverage represented in the present model; accordingly, the kerogen-covered illite system should be interpreted as a controlled end member rather than as a complete representation of natural organic–mineral interfacial heterogeneity.

The calculated water adsorption energy is most negative for Na-smectite at −46.8 kJ/mol, followed by chlorite, illite, and kerogen-covered illite at −40.6, −34.7, and −24.9 kJ/mol, respectively (Figure 5b). Under the same simulation and normalization procedure, these values provide a comparative measure of water–surface interaction strength rather than an adsorption free energy. Their ordering is consistent with the experimental differences in cation exchange capacity and surface oxygen environments and explains why comparable gas-accessible surface areas do not necessarily produce equivalent water retention.

#### 2.3.2. Hydrogen Bond Networks and Molecular Residence Dynamics

The interfacial hydrogen bond network differs markedly among the modeled surfaces (Figure 5c). Na-smectite exhibits the largest water–surface hydrogen bond density, averaging 8.3 nm^−2^, followed by chlorite and illite at 7.1 and 6.2 nm^−2^, respectively. The corresponding value decreases to 3.9 nm^−2^ after partial kerogen coverage. This reduction is accompanied by a greater relative contribution from water–water interactions, indicating weaker direct coupling between the confined water and the underlying mineral surface.

Hydrogen bond persistence further distinguishes the strength of the interfacial restriction. The mean lifetime of water–surface hydrogen bonds is 6.9 ps on Na-smectite, 5.8 ps on chlorite, 4.6 ps on illite, and 2.8 ps on kerogen-covered illite. The residence time of water within the first adsorption layer follows the same sequence, decreasing from 284 ps for Na-smectite to 221 ps for chlorite, 166 ps for illite, and 91 ps for kerogen-covered illite. Na-smectite therefore retains first-layer water through both electrostatic coordination and persistent hydrogen bonding, whereas partial organic coverage weakens direct surface association and facilitates exchange with water farther from the interface.

The common ordering of hydrogen bond lifetime and first-layer residence time is consistent with the experimentally observed importance of charge-related surface properties for water retention. Hydration layer thickness alone, however, does not define an immobile water population. Water molecules in the outer part of the interfacial region exchange more readily with pore-center water, whereas only the innermost population remains associated with the surface for relatively long molecular times. The short T_2_ water identified experimentally should therefore be regarded as a dynamically exchanging ensemble rather than as a permanently fixed monolayer.

#### 2.3.3. Translational–Rotational Dynamics and Molecular Origin of NMR Relaxation

Both translational and rotational motions are retarded within the interfacial region (Figure 5d). The in-plane self-diffusion coefficient of bulk-like pore-center water is approximately 2.18 × 10^−9^ m^2^/s. The corresponding interfacial values decrease to 0.43 × 10^−9^, 0.56 × 10^−9^, 0.73 × 10^−9^, and 1.08 × 10^−9^ m^2^/s for Na-smectite, chlorite, illite, and kerogen-covered illite, respectively. Diffusion perpendicular to the surface is lower than the parallel component for all interfaces, indicating that exchange between the first adsorption layer and the pore center is more restricted than lateral migration along the surface.

The second-order rotational correlation time increases from 2.7 ps for bulk-like water to 18.4 ps on Na-smectite, 15.1 ps on chlorite, 11.6 ps on illite, and 7.0 ps on kerogen-covered illite. Rotational retardation is therefore particularly pronounced on the charged clay surfaces. Because proton dipole–dipole relaxation is sensitive to the persistence of molecular orientations and short-range proton configurations, the prolonged rotational correlation provides a molecular basis for the rapid relaxation of strongly surface-associated water.

The integrated proton–proton dipolar correlation function increases by factors of 3.8, 3.2, 2.6, and 1.7 relative to bulk-like water for Na-smectite, chlorite, illite, and kerogen-covered illite, respectively. The ordering is consistent with the experimental observation that more strongly restricted interfacial water exhibits shorter characteristic T_2_ values. These classical molecular dynamics results describe the intrinsic contribution of molecular restriction to proton dipolar relaxation; they do not include relaxation enhancement arising from electron spins at Fe-related or other paramagnetic centers. The latter contribution is constrained independently by the EPR measurements.

The molecular results therefore explain why neither pore size nor total clay content alone provides a transferable predictor of shale NMR relaxation. Surface charge, hydroxyl-bearing sites, and molecular residence primarily regulate the amount and mobility of interfacial water, whereas the relaxation efficiency additionally depends on the distribution of EPR-detectable magnetic centers. The molecular restriction descriptors derived here are combined with the EPR results in Section 2.4 to evaluate their respective contributions to effective surface relaxivity.

### 2.4. Chemical Controls on Effective Surface Relaxivity

**Figure 6:**
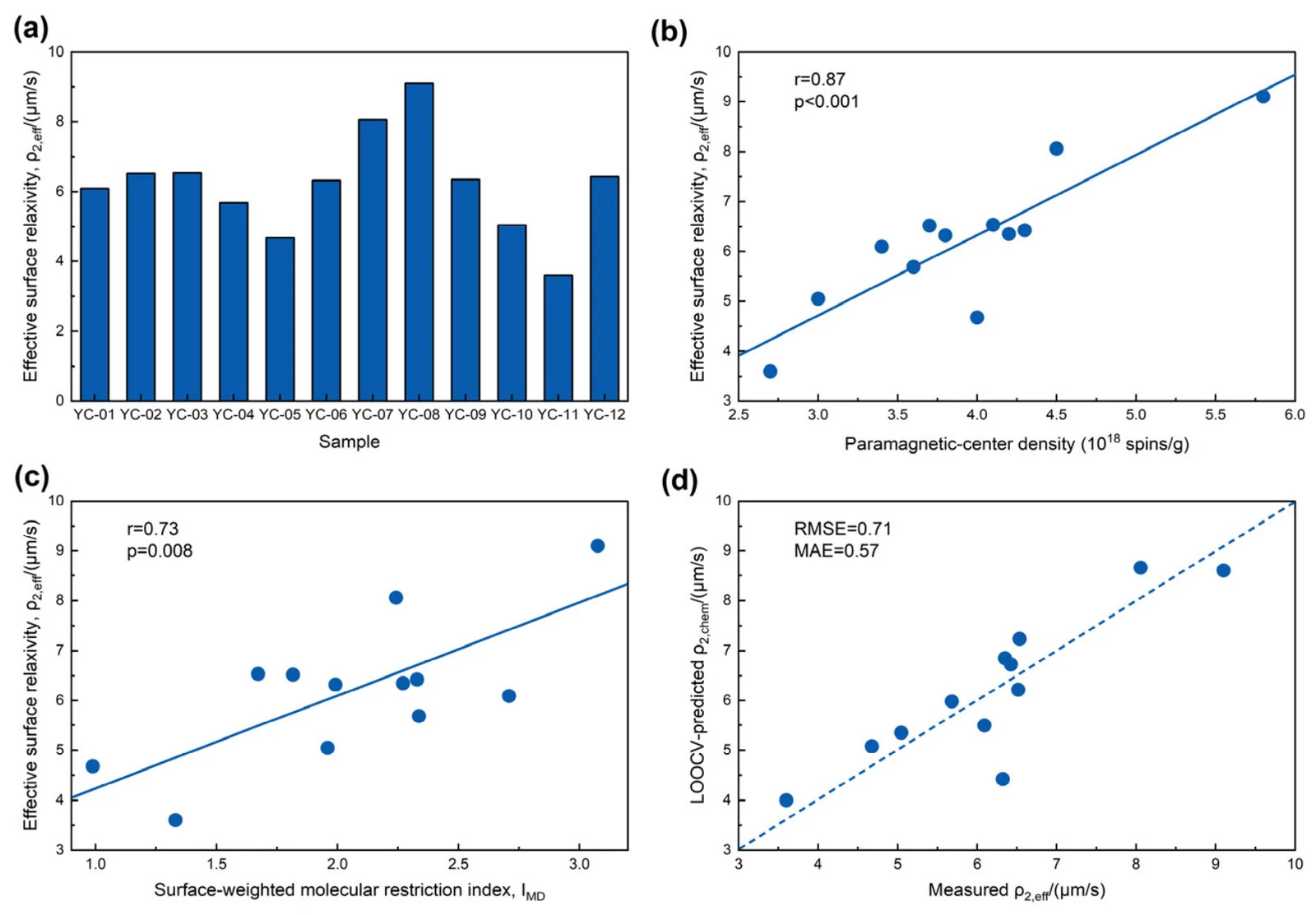
Chemical and molecular controls on effective transverse surface relaxivity: (**a**) sample-specific effective transverse surface relaxivity, *ρ*_2,eff_; (**b**) relationship between *ρ*_2,eff_ and EPR-derived paramagnetic center density; (**c**) relationship between *ρ*_2,eff_ and the surface-weighted molecular restriction index, *I*_MD_; and (**d**) comparison between measured and leave-one-out cross-validated *ρ*_2,eff_. The circles represent individual shale samples, the solid lines in (**b**,**c**) represent linear regression fits, and the dashed line in (**d**) represents the 1:1 relationship.

The effective transverse surface relaxivity, *ρ*_2,eff_, estimated under the matched water-loading condition ranged from 3.6 to 9.1 μm/s, with an average of 6.2 μm/s (Figure 6a). YC-08 exhibited the highest *ρ*_2,eff_, consistent with its large EPR-derived paramagnetic center density and short T_2_ of the strongly surface-associated water population. In contrast, YC-11 showed the lowest relaxivity despite containing appreciable bulk pyrite. YC-07 displayed an intermediate-to-high value, consistent with stronger molecular restriction at its charged clay interfaces despite a lower paramagnetic center density than YC-08. These contrasts demonstrate that sample-specific relaxation efficiency cannot be represented adequately by a single value assigned from bulk lithology. Because the adopted S/V was constrained using gas adsorption-derived surface parameters rather than a direct measurement of water-accessible surface area, the calculated values are treated as operational effective relaxivities under the fast diffusion approximation.

Among the measured interfacial descriptors, EPR-derived paramagnetic center density showed the strongest relationship with *ρ*_2,eff_ (r = 0.87, *p* < 0.001; Figure 6b) and is therefore treated here as the strongest bulk magnetic descriptor rather than as a complete measure of magnetically effective surface sites. Bulk pyrite content did not reproduce the sample ranking in relaxivity, confirming that the abundance of an Fe-bearing mineral alone is not an adequate proxy for the EPR-detectable magnetic environments relevant to proton relaxation [14,15]. Samples with similar mass-normalized spin densities could nevertheless exhibit different relaxation efficiencies if the magnetic centers differ in spatial distribution, clustering, mineral-hosting environment, or accessibility to surface-associated water. Because the present bulk EPR measurement does not resolve these spatial relationships, Npara constrains the abundance of detectable magnetic centers but not their complete proton relaxation effectiveness at the water–solid interface. The surface-weighted molecular restriction index, *I*_MD_, derived from the relative dipolar correlation integrals of the simulated end member interfaces, was also positively related to *ρ*_2,eff_ (*p* = 0.008; Figure 6c). In contrast, neither BET surface area nor total clay content alone showed a significant relationship with relaxivity. These results distinguish the amount of gas-accessible interface from its effective proton relaxation efficiency.

The effects of surface charge and hydroxyl-bearing surface environments were more evident in water retention than in relaxivity itself. Cation exchange capacity and the surface –OH/O–C component were associated primarily with the amount and residence of interfacial water, whereas their direct relationships with *ρ*_2,eff_ were weaker than those of the EPR and molecular restriction descriptors. These associations should not be interpreted as statistically independent effects, because clay abundance and clay assemblage influence both cation exchange capacity and the abundance of exposed oxygen-bearing sites, while TOC may further modify the apparent surface chemistry through partial organic coverage. Given this covariance and the limited 12-sample dataset, the observed relationships are therefore interpreted as evidence for a coupled interfacial control on water retention rather than as a unique partitioning of the contributions from individual compositional variables. Accordingly, charged and hydrophilic surface environments are interpreted as favoring a larger population and stronger molecular restriction of retained water, while the relaxation rate of that water is governed more directly by restricted molecular reorientation and magnetic interactions. This distinction explains why YC-07 retained the largest clay-bound water fraction but did not exhibit the highest surface relaxivity, whereas YC-08 combined substantial water retention with faster relaxation.

To limit overparameterization of the 12-sample dataset, the chemistry-informed model was restricted to two physically defined predictors: the EPR-derived paramagnetic center density, *N*_para_, and the surface-weighted molecular restriction index, *I*_MD_. The standardized two-predictor model explained 86% of the measured relaxivity variation and yielded an adjusted R^2^ of 0.83. Leave-one-out cross-validation produced a root mean square error of 0.71 μm/s and a mean absolute error of 0.57 μm/s, with generally close agreement between the measured and cross-validated predictions (Figure 6d). The model therefore captures complementary magnetic and molecular contributions without introducing additional composition-based predictors into the limited dataset.

Effective surface relaxivity is thus not an intrinsic constant of shale or of an individual clay mineral class. Under the present experimental conditions, it represents a condition-dependent interfacial parameter governed jointly by molecular restriction, EPR-detectable magnetic environments, and exchange between surface-associated and pore-center water. The chemistry-informed model provides a physically constrained alternative to assigning one fixed *ρ*_2_ value throughout a heterogeneous shale interval. Its quantitative calibration remains specific to the adopted S/V treatment, magnetic field, temperature, brine composition, and echo spacing and therefore requires validation before transfer to other laboratory or downhole NMR conditions.

### 2.5. Chemistry-Informed NMR Logging Interpretation and Validation

#### 2.5.1. Core-Scale Comparison with Independent Reference Measurements

The chemistry-informed relaxivity model was first evaluated by comparing the NMR-derived pore structure and clay-bound water parameters with independent gas adsorption and controlled hydration reference measurements. Three interpretation schemes were considered: a fixed relaxivity of 6.2 μm/s, a mineral-corrected relaxivity, and the chemistry-informed variable relaxivity model developed in Section 2.4.

**Figure 7:**
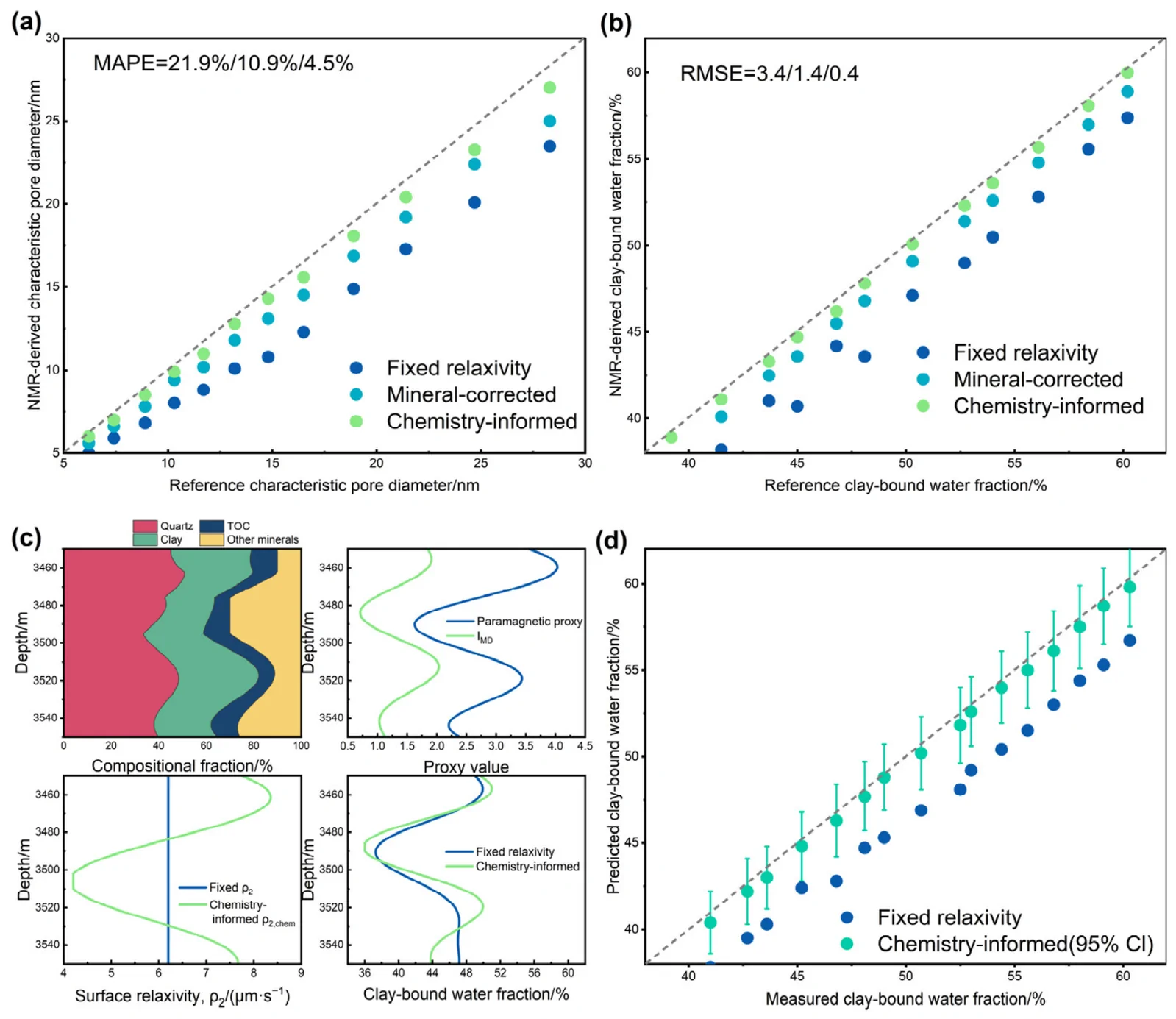
Core- and logging-scale validation of the chemistry-informed NMR interpretation: (**a**) comparison of NMR-derived and reference characteristic pore diameters using fixed, mineral-corrected, and chemistry-informed surface relaxivities; (**b**) comparison of NMR-derived clay-bound water fractions with controlled hydration reference values; (**c**) mineral composition, independently plotted TOC, log-derived magnetic and molecular restriction proxies, depth-dependent surface relaxivity, and clay-bound water interpretation in calibration Well YC-A; and (**d**) blind validation of the clay-bound water fraction in Well YC-B, with error bars representing the 95% confidence intervals. The dashed diagonal lines in (**a**,**b**,**d**) represent the 1:1 relationship between the interpreted or predicted values and the corresponding reference or measured values.

The fixed relaxivity approach reproduced the overall variation in characteristic pore diameter but introduced systematic deviations at the compositional end members (Figure 7a). For samples with relatively strong magnetic relaxation, application of the fixed average relaxivity shifted the NMR-converted pore diameters toward smaller values. The opposite deviation occurred in samples with weaker relaxation efficiency. The mineral-corrected method partly reduced these errors, whereas the chemistry-informed interpretation produced results closest to the independent reference values. The mean absolute percentage error decreased from 21.9% for the fixed relaxivity method to 10.9% for the mineral-corrected method and 4.5% for the chemistry-informed model. The improvement was particularly evident for samples with contrasting EPR responses and molecular restriction characteristics, showing that bulk mineral abundance alone does not fully represent the relaxation efficiency of the exposed interfaces.

A similar improvement was obtained for clay-bound water quantification (Figure 7b). Relative to the controlled hydration reference, the root mean square error of the NMR-derived clay-bound water fraction decreased from 3.4 percentage points using the fixed relaxivity method to 1.4 percentage points using the mineral-corrected method and 0.4 percentage points using the chemistry-informed model. The fixed value produced a systematic underestimation of the reference clay-bound water fraction, whereas incorporation of the magnetic and molecular restriction descriptors substantially reduced this bias. These results indicate that mineral abundance provides only a partial correction because it does not distinguish water-retaining surface environments from EPR-detectable magnetic centers. The chemistry-informed relaxivity preserves this distinction and therefore improves both pore size conversion and clay-bound water partitioning.

Because the same 12 samples were used to establish the relationship between interfacial descriptors and effective relaxivity, Figure 7a,b represents validation against independent measurement references rather than validation using a completely independent core sample set. Nevertheless, the consistent improvement relative to the fixed and mineral-corrected schemes demonstrates that the coupled magnetic and molecular descriptors capture physically meaningful variations in relaxation efficiency.

#### 2.5.2. Field NMR Logging Application and Uncertainty Analysis

The laboratory model was subsequently transferred to two anonymized wells in the Yongchuan Block. Well YC-A was used to establish the depth-dependent logging predictors, whereas Well YC-B was withheld from model calibration and used for blind validation. Before interpretation, the laboratory and logging T_2_ distributions were transformed to a common acquisition reference to reduce differences associated with resonance frequency, echo spacing, and the detectability of very short T_2_ components.

A log-derived magnetic proxy was constructed from elemental Fe abundance and the estimated partitioning of Fe among chlorite, pyrite, and other Fe-bearing mineral phases. This parameter represents a logging-scale proxy for the EPR-detectable magnetic environments rather than a direct downhole measurement of paramagnetic center density. The depth-dependent molecular restriction index was estimated from the log-derived clay mineral and organic matter fractions using the molecular dynamics end member properties and the surface-weighting procedure described in Section 3.7. These two predictors generated a continuously varying *ρ*_2,chem_ profile that differed substantially from the fixed value of 6.2 μm/s over the calibration interval (Figure 7c).

The effect of variable relaxivity was dependent on shale composition. In intervals characterized by a strong magnetic proxy and a high molecular restriction index, the fixed average value underestimated the effective relaxation efficiency and shifted the converted pore size distribution toward smaller values. In quartz-rich intervals with weaker magnetic and molecular restrictions, the same fixed value overestimated relaxation efficiency and expanded the apparent pore size distribution. These opposing effects also propagated into the clay-bound water interpretation. The chemistry-informed correction reduced the compositional biases without modifying the measured total NMR amplitude, indicating that the improvement resulted from a more realistic conversion of relaxation time into pore and water state information rather than from empirical rescaling of porosity.

Blind validation in Well YC-B confirmed that the improvement was retained outside the calibration interval (Figure 7d). The clay-bound water fractions obtained using the fixed relaxivity method were systematically lower than the measured reference values, particularly in the middle and upper parts of the validation range. In contrast, the chemistry-informed predictions were distributed close to the 1:1 relationship, and the measured values were generally encompassed by the corresponding 95% confidence intervals. The field comparison therefore supports the transferability of the coupled magnetic and molecular constraints beyond the core calibration interval.

The principal uncertainty arose from the conversion of elemental and mineralogical logging responses into the magnetic and molecular restriction proxies. In particular, elemental Fe could not always be partitioned uniquely among chlorite, pyrite, and other Fe-bearing phases. Laboratory-to-downhole conversion of frequency and echo-spacing effects introduced an additional uncertainty for water populations approaching the instrumental short T_2_ detection limit. Nevertheless, the differences between the fixed- and chemistry-informed interpretations remained larger than the propagated uncertainty over most of the investigated interval.

The resulting workflow provides a practical route for transferring material-scale interfacial information to NMR logging interpretation without treating surface relaxivity as a uniform lithological constant. The logging predictors should, however, be interpreted as calibrated proxies for the laboratory EPR and molecular descriptors rather than as direct downhole measurements of paramagnetic center density or molecular water dynamics.

## 3. Materials and Methods

### 3.1. Shale Samples and Experimental Design

Twelve core samples, denoted YC-01–YC-12, were collected from the organic-rich Wufeng Formation–lower Longmaxi Formation interval of calibration Well YC-A in the Yongchuan Block, Sichuan Basin. The sampled interval extends from approximately 3450 to 3550 m and was screened to capture the principal mineralogical and organic matter variations observed within this interval. The twelve sampling positions were selected with reference to core observations and available routine mineralogical and TOC information, while maintaining depth coverage and compositional contrasts in quartz–clay proportions, organic matter abundance, and Fe-bearing phases. Accordingly, the selected samples are considered representative of the principal compositional variability of the investigated YC-A interval, rather than of the full mineralogical variability of the Wufeng–Longmaxi Formation at the formation or basin scale. Final inclusion additionally required sufficient intact material for preparation of the NMR plug and sufficient adjacent material for the paired destructive analyses. Candidate core pieces showing obvious drilling-induced or mechanical damage, macroscopic fractures or mineral veins, or insufficient material for paired characterization were excluded during pre-screening; no sample was excluded on the basis of NMR, EPR, gas adsorption, or other analytical results obtained after final sample selection. This strategy increased the compositional contrast available for evaluating the coupled controls on clay-bound water relaxation, although the geological variables were not assumed to be completely independent.

Each core section was divided into adjacent subsamples to minimize the influence of centimeter-scale heterogeneity. A cylindrical plug approximately 25 mm in diameter and 25–30 mm in length was prepared for controlled hydration and NMR measurements. Material collected immediately adjacent to each plug was crushed and divided for whole-rock and clay fraction X-ray diffraction, organic matter characterization, gas adsorption, FTIR, XPS, cation exchange capacity, zeta potential, and EPR measurements. The NMR plugs were not used for destructive characterization. Gravimetric water contents and other mass-normalized parameters were referenced to the vacuum-dry mass obtained under the low-temperature drying conditions described in Section 3.5. Samples YC-01, YC-07, YC-08, and YC-11 were selected for two-dimensional NMR measurements because they represented contrasting combinations of clay composition, surface charge, EPR response, accessible surface area, and organic matter abundance.

The experimental program consisted of four linked stages. First, mineral composition, pore structure, surface functional groups, charge-related properties, and EPR-detectable paramagnetic centers were characterized to define the geometric, chemical, and magnetic properties of the shale interfaces. Second, all 12 NMR plugs were equilibrated sequentially under dry, 33%, 75%, and 97% relative humidity conditions, followed by vacuum saturation, and were analyzed using one-dimensional T_2_ measurements. Two-dimensional T_1_–T_2_ measurements were additionally conducted on the four representative samples. The progressive hydration design provided an experimental basis for distinguishing strongly surface-associated water from multilayer and less restricted pore water without relying solely on an externally imposed universal T_2_ cutoff.

Third, molecular dynamics simulations were performed for Na-smectite, chlorite, illite, and kerogen-covered illite interfaces to quantify water density layering, adsorption strength, hydrogen bonding, molecular residence, translational diffusion, rotational reorientation, and proton–proton dipolar correlation. The Na-smectite model was used to represent the strongly hydratable smectitic component of the mixed-layer illite/smectite identified in the shale samples. The end member simulation results were subsequently combined with the sample-specific mineral and organic compositions using the weighting procedure described in Section 3.7.

Finally, the effective transverse surface relaxivity estimated under the matched water-loading condition and the adopted fast diffusion approximation was related to the EPR-derived paramagnetic center density and the surface-weighted molecular restriction index. Model stability was evaluated by leave-one-out cross-validation across the 12 core samples. The resulting chemistry-informed relaxivity model was transferred to the 3450–3550 m logging interval of Well YC-A after laboratory-to-downhole acquisition correction. A separate interval from 3610 to 3670 m in Well YC-B, containing 16 depth-matched reference points excluded from model development, was used for blind validation. This workflow maintained a clear separation among interfacial mechanism characterization, core-scale model calibration, and logging-scale validation.

### 3.2. Instruments and Software

Whole-rock and clay fraction mineralogy were determined using a D8 ADVANCE X-ray diffractometer (Bruker AXS, Karlsruhe, Germany). Total organic carbon was measured using a Vario TOC cube analyzer (Elementar Analysensysteme GmbH, Langenselbold, Germany), and solid bitumen reflectance was determined using a DM4 P reflected-light microscope equipped with an MSP 200 photometer (Leica Microsystems, Wetzlar, Germany). Low-pressure N_2_ and CO_2_ adsorption measurements were performed using an ASAP 2460 surface area and porosimetry analyzer (Micromeritics Instrument Corporation, Norcross, GA, USA). Surface functional groups and surface elemental chemical states were characterized using a Nicolet iS50 Fourier transform infrared spectrometer and a K-Alpha X-ray photoelectron spectrometer, respectively (Thermo Fisher Scientific, Waltham, MA, USA). Zeta potential was measured using a Zetasizer Nano ZS analyzer (Malvern Panalytical, Malvern, UK). Electron paramagnetic resonance spectra were acquired using an EMXplus spectrometer (Bruker BioSpin, Rheinstetten, Germany), and low-field time-domain NMR measurements were conducted using a GeoSpec 12 MHz rock-core analyzer (Oxford Instruments, Abingdon, UK).

XRD phase identification and Rietveld refinement were performed using DIFFRAC.EVA V8 (Bruker AXS, Karlsruhe, Germany) and DIFFRAC.TOPAS V7 (Bruker AXS, Karlsruhe, Germany), respectively. FTIR, XPS, EPR, and NMR data were processed using OMNIC 9.16 (Thermo Fisher Scientific, Waltham, MA, USA), Avantage V6 (Thermo Fisher Scientific, Waltham, MA, USA), Xepr 2.6b (Bruker BioSpin, Rheinstetten, Germany), and NMR ProLab (Green Imaging Technologies Inc., Fredericton, NB, Canada; official software information accessed on 10 September 2026), respectively. Atomistic interface models were constructed and optimized using BIOVIA Materials Studio 2024, and classical molecular dynamics simulations were performed using the Forcite Plus module within BIOVIA Materials Studio 2024. Molecular dynamics trajectories were further analyzed using custom Python 3.11 scripts to calculate water density distributions, hydrogen bond lifetimes, molecular residence times, diffusion coefficients, rotational correlation functions, and relative proton–proton dipolar correlation integrals. The experimental data-processing software was selected for its direct compatibility with the corresponding instrument outputs and its established functions for the required spectral, diffraction, and relaxation analyses. BIOVIA Materials Studio and Forcite Plus were chosen because they provide an integrated platform for constructing chemically defined mineral–water and organic-covered interfaces and performing classical molecular dynamics simulations under a consistent force field framework. Custom Python scripts were additionally used for trajectory-derived quantitative analyses to ensure that identical computational definitions and processing procedures were applied across all simulated interfaces.

### 3.3. Mineralogical, Pore Structural, and Surface Chemical Characterization

Unless otherwise specified, hydrochloric acid (HCl), ethylene glycol, ammonium acetate (NH_4_OAc), ethanol, potassium chloride (KCl), sodium chloride (NaCl), magnesium chloride (MgCl_2_), and potassium sulfate (K_2_SO_4_) were obtained from Sinopharm Chemical Reagent Co., Ltd. (Shanghai, China). Powdered aliquots collected immediately adjacent to the NMR plugs were used to maintain correspondence among the mineralogical, pore structural, surface chemical, and relaxation measurements. For whole-rock X-ray diffraction, the samples were ground to <75 μm and scanned over a 2θ range of 3–70° using Cu Kα radiation at 40 kV and 40 mA, with a step interval of 0.02°. Mineral abundances were quantified by Rietveld refinement and normalized to 100 wt. The <2 μm clay fraction was separated by sedimentation and prepared as oriented mounts. Air-dried, ethylene glycol-solvated for 24 h, and 550 °C-heated for 2 h, patterns were obtained to distinguish illite, mixed-layer illite/smectite, chlorite, and kaolinite. The relative clay mineral abundances were calculated from the corrected integrated areas of the diagnostic basal reflections and normalized to the total clay mineral fraction.

Total organic carbon was determined after carbonate removal using 10 wt% HCl. The acid-treated residues were repeatedly rinsed with deionized water to neutrality, dried at 60 °C, and analyzed on a dry mass basis. Solid bitumen reflectance was measured on polished whole-rock blocks under oil immersion using monochromatic light at 546 nm. Measurements were restricted to homogeneous post-oil solid bitumen domains; particle margins, mineral inclusions, polishing relief, oxidation rims, and optically heterogeneous regions were excluded. At least 30 valid random reflectance measurements from multiple particles were obtained for each sample and averaged as *BR*_o_. Because primary vitrinite is absent from the investigated marine shale, the equivalent vitrinite reflectance was calculated as

(1) $$R_{o,\mathrm{eq}}=1.125BR_{o}-0.2062$$

where *BR*_o_ is the measured solid-bitumen random reflectance and *R*_o,eq_ is the equivalent vitrinite reflectance.

For gas adsorption measurements, the samples were crushed to 60–80 mesh and degassed under vacuum at 110 °C for 12 h. N_2_ adsorption–desorption isotherms were measured at 77 K over a relative pressure range of P/P_0_ = 0.001–0.995. Specific surface area was calculated using the Brunauer–Emmett–Teller method, with the fitting interval selected according to the Rouquerol consistency criteria. The N_2_-accessible pore volume was estimated from the adsorption amount near P/P_0_ = 0.995, and the mesopore size distribution was derived from the adsorption branch using a non-local density functional theory model with a mixed slit/cylindrical pore kernel. CO_2_ adsorption was measured at 273 K to characterize micropores, particularly ultramicropores that may be kinetically restricted to N_2_ at 77 K. The corresponding micropore surface area and volume were calculated using an appropriate density functional theory kernel. All surface area and pore volume parameters were reported relative to the vacuum-dry sample mass. These parameters were treated as gas probe-accessible pore structure descriptors and were not directly equated with the complete water-accessible surface area or S/V under the NMR conditions.

Fourier transform infrared spectra were acquired in attenuated total reflectance mode over 4000–400 cm^−1^ at a resolution of 4 cm^−1^ by averaging 32 scans. Atmospheric compensation and baseline correction were applied before comparison, and each spectrum was normalized to the Si–O stretching band near 1030 cm^−1^. The normalized spectra were used to compare relative variations in surface functional environments rather than absolute functional group concentrations.

X-ray photoelectron spectra were obtained using monochromatic Al Kα radiation. Survey spectra were collected over 0–1350 eV, followed by high-resolution scans of the C 1s, O 1s, Si 2p, Al 2p, and Fe 2p regions. Binding energies were calibrated to the adventitious carbon C 1s peak at 284.8 eV. High-resolution spectra were fitted after Shirley background subtraction using constrained Gaussian–Lorentzian line shapes. The robustness of the O 1s deconvolution was evaluated by repeated refitting with independently varied initial peak positions and widths while retaining the same component assignments and physical constraints; the resulting component area fractions varied by no more than 1.5 percentage points, which was taken as the fitting uncertainty. The O 1s envelope was resolved into lattice oxygen, a combined surface –OH/O–C component, and a high-binding energy component assigned to weakly bound interfacial oxygen species, including molecularly adsorbed water. Because these oxygen environments may overlap, the O 1s assignments were interpreted jointly with the FTIR, C 1s, and mineralogical results.

Cation exchange capacity was measured by ammonium-acetate saturation at pH 7.0. Approximately 1.0 g of powdered sample was equilibrated with 1 mol/L NH_4_OAc for 24 h, washed with ethanol to remove excess electrolyte, and subsequently treated with 1 mol/L KCl to displace the adsorbed NH_4_^+^. The released ammonium was quantified by distillation and acid titration, and the cation exchange capacity was expressed as cmol(+)·kg^−1^ relative to the vacuum-dry sample mass.

For zeta-potential measurements, the powders were dispersed at 0.05 wt% in 10 mmol/L NaCl solution, adjusted to pH 7.0 ± 0.1, ultrasonicated for 10 min, and equilibrated for 2 h at 25 °C. Electrophoretic mobility was converted to zeta potential using the Smoluchowski approximation. Three independently prepared suspensions were measured for each sample, and the mean value was used in the subsequent correlation and surface relaxivity analyses.

### 3.4. Paramagnetic Center Characterization

Continuous-wave X-band electron paramagnetic resonance measurements were performed on powdered aliquots collected adjacent to the NMR plugs. The samples were dried under vacuum at 60 °C, passed through a 100-mesh sieve, and packed into quartz EPR tubes at a constant filling height to minimize differences in cavity loading. Spectra were acquired at 298 ± 1 K over a magnetic field range of 100–500 mT, using a microwave frequency of approximately 9.40 GHz, a modulation frequency of 100 kHz, and a modulation amplitude of 0.10 mT. The microwave power was fixed at 2.0 mW after preliminary power saturation tests confirmed that the principal signals remained in the nonsaturated regime. Four scans were accumulated for each spectrum. Receiver gain and cavity quality factor were recorded for subsequent intensity correction.

The magnetic field scale and g values were calibrated using a DPPH standard (Sigma-Aldrich, St. Louis, MO, USA) with g = 2.0036. After baseline correction, the first-derivative spectra were analyzed using Xepr software. The narrow signal near g = 2.003 was assigned predominantly to persistent organic radicals, whereas broader Fe-related contributions occurred around g ≈ 2.0 and g ≈ 4.3. The latter feature was assigned mainly to isolated high-spin Fe^3+^ in distorted mineral coordination environments. Because the organic radical and Fe-related contributions overlap near g = 2.0, their relative components were evaluated by constrained derivative line-shape fitting rather than by direct peak-height comparison. The spectral assignments were interpreted jointly with the XRD-derived pyrite and clay mineral contents and the Fe 2p XPS results.

The effective EPR-detectable paramagnetic center density was quantified from the double integral of the complete baseline-corrected spectrum using a calibrated weak-pitch spin standard (Bruker BioSpin, Rheinstetten, Germany). The mass-normalized spin density was calculated as

(2) $$N_{\mathrm{para}}=\frac{DI_{s}}{DI_{\mathrm{ref}}}\frac{G_{\mathrm{ref}}}{G_{s}}{\left(\frac{P_{\mathrm{ref}}}{P_{s}}\right)}^{1/2}\frac{Q_{\mathrm{ref}}}{Q_{s}}\frac{N_{\mathrm{ref}}}{m_{s}}$$

where *DI* is the double-integrated EPR intensity, *G* is the receiver gain, *P* is the microwave power, *Q* is the cavity quality factor, *N*_ref_ is the known number of spins in the reference standard, and *m*_s_ is the dry sample mass. Subscripts “s” and “ref” denote the shale sample and reference standard, respectively. The calculated *N*_para_ values were expressed as spins/g.

Three independently packed aliquots were measured for each sample, and the mean spin density was used in the relaxivity analysis. The relative standard deviation was maintained below 5%. The reported *N*_para_ represents the total population of EPR-detectable centers under the specified experimental conditions; it should therefore be regarded as an effective magnetic site descriptor rather than a direct measure of only surface-accessible Fe^3+^. Its relevance to proton relaxation was assessed through its relationship with the geometric mean T_2_ of bound water and the effective transverse surface relaxivity.

### 3.5. Controlled Hydration and Multidimensional NMR Measurements

The cylindrical shale plugs were dried under vacuum at 60 °C for 48 h and subsequently maintained under the same conditions until the mass change was below 0.05% over 24 h. A dry-state NMR spectrum was recorded for each plug to characterize the detectable residual hydrogen signal from solid organic matter, structural hydroxyls, and other rapidly relaxing components. Controlled hydration was then conducted at 25 ± 0.2 °C in sealed desiccators containing saturated MgCl_2_, NaCl, and K_2_SO_4_ solutions, which maintained relative humidities of approximately 33%, 75%, and 97%, respectively. The plugs were equilibrated sequentially from low to high relative humidity along the adsorption pathway. Constant mass was used as the equilibration criterion; equilibrium was considered reached when the mass change was below 0.10% over two consecutive 24 h intervals, which generally required 7–12 d at each humidity level. The gravimetric water content was calculated as

(3) $$w_{H_{2}O}=\frac{m_{h}-m_{d}}{m_{d}}\times 100\%$$

where *m*_h_ and *m*_d_ are the hydrated and dry sample masses, respectively. Water uptake at 97% relative humidity was used as the gravimetric bound water capacity because liquid capillary condensation remained limited under this condition.

After completion of the humidity-controlled sequence, the plugs were vacuum saturated with 0.5 mol/L NaCl solution. The samples were evacuated at an absolute pressure below 5 kPa for 12 h, after which the brine was introduced without breaking the vacuum. Saturation was completed at 10 MPa for 24 h. The plug surfaces were then gently blotted, immediately weighed, sealed in fluorinated polymer film, and transferred to the NMR probe. An additional matched water-loading condition of 4.00 ± 0.05 wt% was prepared for cross-sample comparison by microgravimetric addition of the same NaCl solution to the vapor-equilibrated plugs, followed by sealed equilibration for 72 h. This matched-loading condition was used to compare relaxation behavior while minimizing differences caused by total water content.

All NMR measurements were performed at 25 ± 0.2 °C using a proton resonance frequency of 12 MHz. One-dimensional T_2_ distributions were acquired using the Carr–Purcell–Meiboom–Gill sequence with an echo spacing of 0.08 ms, 16,000 echoes, a recycle delay of 5 s, and 64 accumulated scans. The instrumental dead time was approximately 40 μs. The dry-state contribution was corrected for receiver gain and sample mass and removed before quantitative interpretation of the hydrated response. The T_2_ distributions were obtained by non-negative Tikhonov-regularized inversion using 120 logarithmically spaced bins between 0.01 and 1000 ms. The regularization coefficient was selected using generalized cross-validation and was held constant when comparing the different hydration states of the same sample.

The amplitude-weighted geometric mean relaxation time of a selected water population was calculated as

(4) $$T_{2,g}=\exp\left(\frac{\sum_{i=1}^{n}A_{i}\ln T_{2,i}}{\sum_{i=1}^{n}A_{i}}\right)$$

where *A_i_* is the inverted signal amplitude associated with relaxation time *T*_2,*i*_. The geometric mean T_2_ reported for interfacial water was evaluated at the matched water loading of 4.0 wt%, thereby reducing the influence of differences in total pore filling among samples.

Two-dimensional T_1_–T_2_ measurements were performed on YC-01, YC-07, YC-08, and YC-11 using an inversion recovery CPMG sequence. Thirty-two logarithmically spaced inversion times between 0.2 ms and 5 s were used, followed by a CPMG echo train with an echo spacing of 0.08 ms and 4096 echoes. Sixteen scans were accumulated for each inversion time. The two-dimensional distributions were reconstructed by non-negative regularized inverse Laplace transformation on logarithmic T_1_ and T_2_ grids. All four maps were inverted using the same grid, regularization strategy, and intensity normalization to permit direct comparison.

The clay-bound water region was identified by jointly considering the progressive hydration spectra and the minima separating the two principal populations in the T_1_–T_2_ maps. Under the present acquisition conditions, strongly surface-associated water was concentrated mainly at T_2_ < 1.6 ms and T_1_ < 85 ms. These limits were derived from the controlled hydration dataset rather than adopted from a universal shale cutoff. The T_2_ = 1.6 ms boundary was subsequently applied to the saturated one-dimensional spectra of all 12 samples. The clay-bound water fraction was calculated as

(5) $$f_{\mathrm{CBW}}=\frac{\sum_{T_{2,i}<1.6\;\mathrm{ms}}A_{i}}{\sum_{i=1}^{n}A_{i}}\times 100\%$$

For the representative two-dimensional measurements, the integrated intensity within the corresponding T_1_–T_2_ region was additionally used to verify the one-dimensional partitioning. Duplicate acquisitions were conducted for the one-dimensional T_2_ measurements at each hydration state and for the representative two-dimensional T_1_–T_2_ measurements. The relative deviations in the geometric mean T_2_ and T_1_ values of the resolved water populations were below 3.0% and 4.0%, respectively, while the deviation in total NMR amplitude remained below 3.0%. These repeatability ranges were taken as the experimental uncertainties under identical acquisition and inversion conditions, and the averaged values were used in the subsequent analyses.

### 3.6. Molecular Dynamics Simulation

Atomistic models of Na-smectite, chlorite, illite, and kerogen-covered illite were constructed in BIOVIA Materials Studio 2024 as selected end members of the clay-associated and organic-covered interfaces most directly relevant to the clay-bound water relaxation mechanism examined in this study. Quartz and carbonate surfaces were not included because the molecular simulations were deliberately restricted to this clay-focused end member set; their omission should not be interpreted as implying negligible water–surface interactions. Given the substantial quartz abundance and subordinate carbonate content of the samples, these mineral surfaces may also contribute to the overall water relaxation response, and their exclusion therefore represents a limitation of the present molecular model set. The kerogen-covered illite configuration was selected because illite is the dominant clay mineral and was used as a controlled organic-masking end member at 35% areal coverage rather than as a complete representation of natural kerogen–mineral heterogeneity. The Na-smectite model was used as an end member for the strongly hydratable smectitic component of mixed-layer illite/smectite rather than as a direct representation of the complete mixed-layer structure. Mineral unit cells were expanded parallel to the basal plane to obtain surface dimensions of approximately 5.2 × 5.4 nm, and two opposing slabs were separated by a 4.0 nm water-filled slit pore. Representative isomorphic substitutions and charge-compensating Na^+^ or K^+^ ions

(6) $$C_{2}(t)=\langle P_{2}\left[\mu (0)\cdot \mu (t)\right]\rangle$$

were introduced according to the structural charge of each clay mineral. For the organic-covered model, an overmature type-II kerogen fragment was placed on the illite surface at an areal coverage of approximately 35%, leaving both mineral-exposed and organic-covered regions accessible to water. The pore fluid contained 0.5 mol/L NaCl, consistent with the brine used in the NMR experiments.

Classical molecular dynamics simulations were performed using the Forcite Plus module with the COMPASS III force field. COMPASS III was applied consistently to both the clay mineral phases and the kerogen organic phase to maintain a unified description of bonded and nonbonded interactions across the mineral–organic–water interfaces. Water molecules and Na^+^/Cl^−^ ions were parameterized within the same COMPASS III force field framework; no separately parameterized SPC/E or TIP3P water model was introduced. Electrostatic interactions were evaluated using Ewald summation, and van der Waals interactions were treated using an atom-based cutoff of 1.25 nm. Following geometry optimization and annealing, each system was equilibrated for 1 ns in the NVT ensemble at 298 K and subsequently subjected to a 10 ns production run with a time step of 1 fs. Equilibration was verified by monitoring the stabilization of temperature and potential energy without systematic drift, while production convergence was assessed from the stabilization of cumulative and block-averaged interfacial descriptors, including water density distributions, hydrogen bond statistics, residence behavior, and diffusion coefficients. The innermost structural layer of each mineral slab was position-restrained to maintain the crystalline framework, whereas surface atoms, exchangeable ions, water molecules, and kerogen atoms remained mobile. Three simulations with independently assigned initial velocities were conducted for each interface.

The production trajectories were analyzed to obtain water density profiles normal to the interface, effective hydration layer thickness, water adsorption energy, water–surface hydrogen bond density and lifetime, and residence time within the first adsorption layer. Translational mobility was evaluated from the mean-square displacement components parallel and perpendicular to the interface. The corresponding diffusion coefficients were determined from the linear diffusive region of the trajectories using the Einstein relation. Rotational restriction was characterized using the second-order orientational autocorrelation function of the water molecular dipole,

(7) $$C_{2}(t)=\langle P_{2}\left[\mu (0)\cdot \mu (t)\right]\rangle$$

where *P*_2_ is the second-order Legendre polynomial, and **μ** is the unit vector along the water dipole. The rotational correlation time was obtained by integrating *C*_2_(*t*) over the selected correlation interval.

Intermolecular proton–proton dipolar correlation functions were additionally calculated from the production trajectories. Their integrals were normalized to the corresponding value for pore-center water to obtain the relative dipolar correlation integral for each interface. This parameter was used as a molecular descriptor of the intrinsic relaxation enhancement associated with restricted translation and reorientation. The same spatial definitions, trajectory intervals, and analysis procedures were applied to all four interface systems to preserve comparability.

The relative dipolar correlation integrals of the simulated end members were subsequently combined with the sample-specific surface composition weights to calculate the molecular restriction index, *I*_MD_, as described in Section 3.7. The classical molecular dynamics calculations did not include electron spin interactions and therefore did not directly quantify paramagnetic relaxation enhancement. Magnetic contributions were constrained independently using the EPR-derived paramagnetic center density.

### 3.7. Chemistry-Informed Surface Relaxivity Model and Logging Validation

The effective transverse surface relaxivity of each core sample was estimated under the matched water-loading condition using the surface relaxation relation

(8) $$\frac{1}{T_{2,g}}=\frac{1}{T_{2,b}}+\rho_{2,\mathrm{eff}}{\left(\frac{S}{V}\right)}_{\mathrm{eff}}$$

where *T*_2,g_ is the geometric mean relaxation time of the interfacial water population, *T*_2,b_ is the relaxation time of the bulk 0.5 mol/L NaCl solution measured at the same temperature and resonance frequency, and (S/V)_eff_ is the effective surface-to-water-volume descriptor adopted for the calculation. The surface contribution was constrained using the combined N_2_- and CO_2_-adsorption parameters, whereas the water-filled volume was determined from the gravimetric water content and NMR signal amplitude. Because gas adsorption and saline water do not necessarily access identical pore surfaces, (S/V)_eff_ was treated as an operational, gas adsorption-constrained descriptor rather than as the complete water-accessible S/V. Accordingly, the calculated *ρ*_2,eff_ values represent effective interfacial parameters under the adopted surface relaxation approximation.

The molecular restriction index, *I*_MD_, was calculated by weighting the relative proton–proton dipolar correlation integral of each simulated interface according to its contribution to the represented shale surface:

(9) $$I_{\mathrm{MD}}=\sum_{j=1}^{n}w_{j}I_{j},\;w_{j}=\frac{f_{j}S_{j}}{\sum_{k=1}^{n}f_{k}S_{k}}$$

Here, *I*_j_ is the relative dipolar correlation integral of interface *j*, normalized to pore-center water; *f*_j_ is the sample-specific compositional fraction assigned to that interface class from the measured mineralogical and organic matter composition; and *S*_j_ is its corresponding specific surface area contribution in the surface-weighting scheme. Thus, *w*_j_ is the normalized product *f*_j_*S*_j_, rather than an independently fitted coefficient. Illite, the hydratable smectitic contribution of mixed-layer illite/smectite, chlorite, and organic-covered mineral surfaces were represented by the illite, Na-smectite, chlorite, and kerogen-covered illite end members, respectively. The *I*_j_ values were obtained independently from the molecular dynamics trajectories and were not optimized against the measured NMR relaxivity. For fixed end member *I*_j_ and *S*_j_, the local sensitivity of the index to a compositional fraction *f*_m_ follows $\partial I_{\mathrm{MD}}/\partial f_{m}=S_{m}(I_{m}-I_{\mathrm{MD}})/\sum_{k}f_{k}S_{k}$; therefore, mineralogical changes toward interfaces with larger-than-average dipolar correlation integrals increase *I*_MD_, whereas increasing the contribution of interfaces with smaller *I*_j_ decreases it. Given the end member sequence Na-smectite (3.8) > chlorite (3.2) > illite (2.6) > kerogen-covered illite (1.7), the index is consequently most responsive to shifts in the relative surface contributions of strongly restricting smectitic/chloritic interfaces versus organic-covered interfaces, rather than to bulk mineral abundance alone. This index describes the relative intrinsic contribution of interfacial molecular restriction and does not include electron spin-induced paramagnetic relaxation.

To limit overparameterization of the 12-sample dataset, the chemistry-informed model retained only two predictors: the effective EPR-detectable paramagnetic center density, *N*_para_, and the surface-weighted molecular restriction index, *I*_MD_. Both predictors were standardized as

(10) $$z(x)=\frac{x-\bar{x}}{s_{x}}$$

$$\rho_{2,\mathrm{chem}}=6.15+1.42z(N_{\mathrm{para}})+0.76\;z(I_{\mathrm{MD}})$$

where $\bar{x}$ and *s_x_* are the sample mean and standard deviation, respectively. The model was expressed in the general form

(11) $$\rho_{2,\mathrm{chem}}=\beta_{0}+\beta_{1}z(N_{\mathrm{para}})+\beta_{2}z(I_{\mathrm{MD}})$$

where *ρ*_2,chem_ is expressed in μm/s and *β*_0_, *β*_1_, and *β*_2_ are coefficients estimated by ordinary least squares. The final coefficients were obtained from the same unified 12-sample dataset used to generate Figure 6. Model stability was evaluated by leave-one-out cross-validation. Predictor standardization and coefficient estimation were repeated within each training fold to prevent information leakage. Performance was quantified using adjusted R^2^, root mean square error, and mean absolute error.

For logging application, Well YC-A was used to establish the core–log calibration, whereas Well YC-B was withheld for blind validation. Laboratory and downhole T_2_ distributions were transformed to a common reference of 12 MHz and 0.08 ms echo spacing using depth-matched calibration relationships derived from Well YC-A. A log-derived magnetic proxy was constructed from elemental Fe after partitioning the Fe signal among chlorite-, pyrite-, and residual Fe-bearing contributions using the mineral-log constraints. At the 12 core-matched depths in Well YC-A, the phase-associated Fe contributions were related to the laboratory EPR-derived *N*_para_ values using the calibration form *M*_mag_ = *a*_0_ + *a*_chl_*F*_Fe,chl_ + *a*_pyr_*F*_Fe,pyr_ + *a*_oth_*F*_Fe,oth_, where the coefficients were estimated exclusively from the YC-A core–log pairs; the resulting relationship was subsequently applied without refitting to the continuous logging data and to Well YC-B. The calibrated quantity was treated as an EPR-equivalent magnetic proxy rather than as a direct downhole measurement of spin density. The log-derived molecular restriction proxy was calculated from the clay mineral and organic matter fractions using the same end member mapping and normalized surface-weighting framework applied to the core samples. These proxies were converted to the laboratory-equivalent standardized predictor scales before being inserted into the calibrated relaxivity model. For robustness, simpler formulations based on total elemental Fe alone and pyrite-associated Fe alone were also evaluated using the same YC-A calibration and leave-one-out procedure; both produced larger cross-validated residuals relative to laboratory *N*_para_, and the phase-partitioned formulation was therefore retained.

The resulting depth-dependent *ρ*_2,chem_ profile was used for NMR pore size conversion and clay-bound water partitioning. Three interpretation schemes were compared: a fixed relaxivity of 6.2 μm/s, a bulk mineral-corrected relaxivity based only on mineral composition, and the chemistry-informed variable relaxivity model. Core-scale evaluation used gas adsorption-derived characteristic pore diameters and controlled hydration clay-bound water fractions as reference measurements. Blind validation in Well YC-B used 16 depth-matched reference points that were excluded from model fitting.

Uncertainty was evaluated by Monte Carlo propagation through the complete proxy-to-interpretation workflow. In each realization, elemental Fe and mineral fractions were perturbed according to their measurement and core–log calibration uncertainties; the partitioning of Fe among the represented mineral phases and the resulting magnetic proxy were recalculated, and uncertainty in the core–EPR calibration coefficients was propagated together with that of the molecular dynamics end member parameters and relaxivity model residuals. The corresponding *I*_MD_, *ρ*_2,chem_, pore size conversion, and clay-bound water fraction were then recalculated. Ten thousand realizations were generated for each depth point, and the 2.5th and 97.5th percentiles of the resulting distributions were reported as the 95% confidence interval. Prediction accuracy was assessed using R^2^, RMSE, MAE, and mean absolute percentage error.

## 4. Conclusions

This study clarifies the interfacial chemical and molecular controls on clay-bound water relaxation in organic-rich shale and demonstrates that transverse surface relaxivity cannot be represented adequately by a constant determined solely from pore geometry or bulk mineral composition. Three principal conclusions are drawn.

First, the NMR response of clay-bound water depends strongly on its interfacial physicochemical state. Under the present conditions of 12 MHz resonance frequency, 0.5 mol/L NaCl brine, 25 ± 0.2 °C, and 0.08 ms echo spacing, strongly surface-associated water was concentrated mainly at T_2_ < 1.6 ms and T_1_ < 85 ms. These limits represent experimentally defined operational boundaries rather than universal shale water cutoffs. Samples with comparable gas-accessible surface areas nevertheless exhibited substantially different short T_2_ responses, and the effective transverse surface relaxivity ranged from 3.6 to 9.1 μm/s. The calculated values should therefore be regarded as condition-dependent effective interfacial parameters associated with the adopted S/V treatment and surface relaxation approximation.

Second, water retention and proton relaxation efficiency are controlled by related but distinguishable interfacial properties. Cation exchange capacity, charge-related surface environments, and hydroxyl- and oxygenated carbon-associated sites were more closely associated with water uptake, molecular residence, and the degree of interfacial restriction, although these descriptors partly covary with clay assemblage and organic matter abundance. Molecular simulations further show that Na-smectite, chlorite, illite, and kerogen-covered illite produce distinct water density layering, adsorption strength, hydrogen bond persistence, residence time, translational diffusion, and rotational reorientation. Partial organic coverage reduces the exposure of hydrophilic mineral sites and weakens direct water–mineral association. The relaxation efficiency of the retained water is governed more directly by molecular-motion restriction and EPR-detectable paramagnetic centers, rather than by total clay or bulk pyrite abundance alone.

Third, the EPR-derived paramagnetic center density and the surface-weighted molecular restriction index were integrated into a chemistry-informed variable relaxivity model. The two-predictor model yielded an adjusted R^2^ of 0.83, with leave-one-out cross-validation RMSE and MAE values of 0.71 and 0.57 μm/s, respectively. At the core scale, the chemistry-informed interpretation reduced the mean absolute percentage error of characteristic pore diameter from 21.9% for the fixed relaxivity method to 4.5%, while the RMSE of the clay-bound water fraction decreased from 3.4 to 0.4 percentage points. Transfer through calibrated logging-scale magnetic and molecular restriction proxies also improved the agreement with the blind-validation data from Well YC-B.

Overall, surface relaxivity in organic-rich shale is an emergent interfacial property arising from the coupled effects of water-retaining surface chemistry, molecular restriction, and EPR-detectable magnetic environments. The proposed framework provides a mechanistic basis for improving NMR analysis of chemically heterogeneous shale materials, while the logging application serves as a scale validation step. Its quantitative coefficients require recalibration when the mineral–organic composition, brine chemistry, temperature, magnetic field, echo spacing, or adopted surface-to-volume treatment changes substantially. Future laboratory work should combine variable field and variable temperature NMR with high-resolution pore imaging and advanced magnetic characterization to directly constrain water-accessible pore geometry and the spatial relationship between interfacial water and paramagnetic sites, thereby further testing the applicability of the proposed relaxivity framework under broader shale compositions and fluid conditions.

## Data Availability

The data supporting the findings of this study are available within the article and its Supplementary Materials.

## Supplementary Materials

The following supporting information can be downloaded at: https://www.mdpi.com/article/10.3390/molecules31183185/s1, Table S1, Whole-rock mineral composition, clay mineral composition, total organic carbon content, and equivalent vitrinite reflectance of the investigated shale samples; Table S2, N_2_- and CO_2_-adsorption-derived pore structure parameters of the investigated shale samples.
