# Supplementary material for: Interfacial Molecular Mechanisms Governing the NMR Relaxation of Clay-Bound Water in Organic-Rich Shales with Implications for NMR Logging
Source: Molecules. 2026 Sep 10;31(18):3185. doi: 10.3390/molecules31183185 (PMC13609254; doi:10.3390/molecules31183185)
Supplement: Supplementary file 1 [file molecules-31-03185-s001.zip › molecules-4538588-supplementary.pdf]

Supplementary Materials

Table S1. Whole-rock mineral composition, clay-mineral composition, total organic carbon content, and equivalent vitrinite reflectance of the investigated shale samples.

Sample	Quartz (wt%)	Feldspar (wt%)	Calcite (wt%)	Dolomite (wt%)	Pyrite (wt%)	Clay minerals (wt%)	Illite (% clay)	I/S (% clay)	Chlorite (% clay)	Kaolinite (% clay)	TOC (wt%)	$R_{o,eq}$(%)
YC-01	54.0	5.8	6.4	3.1	3.2	27.5	61	17	18	4	5.42	2.72
YC-02	48.3	6.2	7.8	2.9	4.9	29.9	63	15	18	4	4.86	2.66
YC-03	47.9	5.4	8.6	3.7	2.8	31.6	58	20	17	5	5.18	2.78
YC-04	52.2	4.8	5.1	2.6	5.0	30.3	66	13	18	3	4.31	2.63
YC-05	42.5	6.7	9.4	3.6	3.6	34.2	59	19	17	5	3.72	2.69
YC-06	46.2	5.9	8.1	3.2	4.4	32.2	62	16	18	4	4.12	2.61
YC-07	41.4	7.4	10.2	4.8	2.3	33.9	57	22	16	5	3.05	2.75
YC-08	45.5	5.3	7.2	3.5	3.0	35.5	60	18	17	5	4.67	2.58
YC-09	38.1	7.9	11.6	4.7	5.1	32.6	56	21	19	4	2.86	2.81
YC-10	50.4	6.1	6.8	2.9	2.7	31.1	65	14	18	3	3.58	2.67
YC-11	39.9	8.3	12.4	4.2	4.6	30.6	58	19	18	5	2.44	2.64
YC-12	47.9	5.7	8.9	3.1	3.8	30.6	64	15	17	4	5.09	2.73

Notes: Whole-rock mineral contents are expressed relative to the bulk dry sample and were normalized to 100 wt%. Illite, mixed-layer illite/smectite, chlorite, and kaolinite contents are expressed as percentages of the total clay-mineral fraction and were normalized to 100%. I/S denotes mixed-layer illite/smectite; TOC denotes total organic carbon; and $R_{o,eq}$ denotes equivalent vitrinite reflectance.

Table S2. N_2- and CO_2-adsorption-derived pore-structure parameters of the investigated shale samples.

Sample	N_2-BET specific surface area (m^2/g)	N_2-accessible pore volume (cm^3/g)	Dominant mesopore diameter (nm)	CO_2-DFT micropore surface area (m^2/g)	CO_2-DFT micropore volume (cm^3/g)
YC-01	25.6	0.0258	3.6	12.4	0.0049
YC-02	24.3	0.0264	3.8	10.3	0.0043
YC-03	30.8	0.0309	3.4	12.6	0.0051
YC-04	23.5	0.0251	4.1	8.9	0.0038
YC-05	26.1	0.0328	4.3	11.3	0.0046
YC-06	24.7	0.0286	4.0	9.6	0.0040
YC-07	26.9	0.0337	4.6	11.1	0.0047
YC-08	31.6	0.0361	3.5	13.1	0.0053
YC-09	22.4	0.0274	4.2	9.4	0.0041

YC-10	20.8	0.0246	4.0	8.8	0.0037
YC-11	18.9	0.0238	4.4	8.6	0.0039
YC-12	28.7	0.0297	3.7	11.5	0.0046

Note: The N_2- and CO_2-derived parameters represent gas-probe-accessible pore surfaces and volumes. They should not be directly equated with the complete water-accessible surface area or surface-to-volume ratio under the NMR experimental conditions.
